# The effect of diet on the structure of gut bacterial community of sympatric pair of whitefishes (*Coregonus lavaretus*): one story more

**DOI:** 10.7717/peerj.8005

**Published:** 2019-12-03

**Authors:** Mikhail M. Solovyev, Elena N. Kashinskaya, Nickolai A. Bochkarev, Karl B. Andree, Evgeniy Simonov

**Affiliations:** 1 Institute of Systematics and Ecology of Animals, Siberian Branch of Russian Academy of Sciences, Novosibirsk, Russia; 2 Tomsk State University, Tomsk, Russia; 3 Instituto de Investigación y Tecnología Agroalimentarias, San Carlos de la Rapita, Tarragona, Spain; 4 Institute of Environmental and Agricultural Biology, Tyumen State University, Tyumen, Russia

**Keywords:** Oligotrophic lake, Coregonids, Mycoplasma, Feeding habits, Proteobacteria, Stomach microbiota, Microbiota of pyloric caeca

## Abstract

In the *Coregonus lavaretus* complex may be found lacustrine sympatric pairs, which serves as an intriguing model for studying different aspects of fish evolutionary biology. One such sympatric whitefish pair inhabits Teletskoye Lake (West Siberia, Russia) and includes a “large” form (*Coregonus lavaretus pidschian* (Gmelin, 1789)) and a “small” form (*C. l. pravdinellus* (Dulkeit, 1949)). *C. l. pravdinellus* has a narrow trophic specialization and feeds on zooplankton, whereas the diet of *C. l. pidschian* is based on benthic prey. In the present study we aimed to address the question of how the gut microbial community reflects the divergence in diet of a sympatric pair of whitefish. Studied samples included the mucosa and content were collected for cardiac and pyloric stomach, anterior, middle, and posterior intestine, but only mucosa was collected for the pyloric caeca. In addition, water, sediment, macrophyte (environmental microbiota) and invertebrate (microbiota of prey) samples were collected in the same location. The V3–V4 region of the 16S rRNA genes was chosen for microbiome analysis and the software PICRUSt used to estimate the difference functional roles of the microbiota. The number of OTUs and Chao1 index in mucosa and content of cardiac and pyloric stomach were significantly different between whitefish. Significant differences were observed between whitefish for content from different parts of the intestine in terms of OTU number and Chao1 indices, whereas for mucosa from the same parts of intestine these differences were absent. No significant differences were found for diversity estimates of mucosa and content of different parts of the gut (there were a few exceptions) between whitefish. The form of whitefish and the segment of the digestive system were factors with a significant determinative effect on the structure of the microbiota from gut mucosa and content. The most dominant phyla in mucosa and content of cardiac and pyloric stomach was Proteobacteria (57.0–84.0%) for both whitefish. Throughout the intestine of *C. l. pidschian* the dominant phyla in mucosa were Proteobacteria (38.8%) and Firmicutes (15.6%), whereas for *C. l. pravdinellus*–Tenericutes (49.6%) and Proteobacteria (28.1%). For both forms, the phylum Spirochaetes was found in a significant amount (20.0–25.0%) in the mucosa of the posterior intestine. While for the content obtained from anterior, middle and posterior intestines, the dominant bacterial phyla were the same as those described for mucosa from the same parts of the intestine for both whitefish. The bacterial community of the prey and environment was significantly different from bacterial communities found for all parts of the gut mucosa for both whitefish, with the exception of the mucosa of the cardiac stomach. According to PICRUSt the highest level of differences between whitefish at the L3 level were found for the intestinal mucosa (75.3%), whereas the lowest one was registered for stomach content (38.8%).

## Introduction

The enteric bacterial community is a complex and dynamic microbial ecosystem within the gut of animals. These bacterial communities possess multiple different functions and play important roles in various physiological processes including disease prevention for their host (Verschuere et al., 2000; Araújo et al, 2014; Vasemägi, Visse & Kisand, 2017). It has been suggested that symbiotic microorganisms might even be involved in speciation of eukaryotes (Brucker & Bordenstein, 2012). More recently, the role of such symbionts on pre-zygotic isolation was confirmed (reviewed by Shropshire & Bordenstein, 2016). However, the gut microbiome of all organisms is constantly subjected to a plethora of different factors that may have effects on and alter this community (Austin, 2002; Sullam et al., 2012; Clements et al., 2014; Kashinskaya et al., 2017; Kashinskaya et al., 2018). It has been shown that one of the main factors that can determine and modify the qualitative (taxonomic composition) and quantitative (relative abundance of each taxa) composition of gut bacterial community is the host diet (Wu et al., 2010; Sullam et al., 2012; Bolnick et al., 2014a; Bolnick et al., 2014b; Larsen, Mohammed & Arias, 2014; Li et al., 2014a; Li et al., 2014b; Tietjen, 2014; Miyake, Ngugi & Stingl, 2015; Liu et al., 2016; Baldo et al., 2017; Kashinskaya et al., 2015; Kashinskaya et al., 2018).

The aforementioned importance of the gut bacterial communities for the ecology, physiology, and even population differentiation/speciation of their hosts, has fueled the rapid growth of research interest in the topic (Sullam et al., 2015; Gajardo et al., 2016; Sevellec, Derome & Bernatchez, 2018). At the same time there are more than 34,000 known fish species in the world (FishBase ver. 10/2018, http://www.fishbase.org) characterized by different features of morphology (presence/absence of stomach, pyloric caeca, spiral valve, etc.) and physiology (for example, presence/absence of acid stage of digestion) of the digestive system. Additionally, each has their own distinct feeding habits, and inhabits various types of water bodies with different chemical composition and temperature regimes. With such diverse types of influences on the composition of the gut microbial community, the primary drivers of diversity and the mechanisms for establishing stability of the microbial community are indistinct. Of particular value in addressing this issue would be studies focused on sympatric fish species/subspecies (or forms) with ecological niche segregation (Franchini et al., 2014; Sevellec et al., 2014; Sevellec, Derome & Bernatchez, 2018; Belkova et al., 2017). As a rule, such species originated relatively recently from the common ancestor by adaptation to different trophic niches (Bernatchez, 2004; Barluenga et al., 2006; Elmer et al., 2010). Thus, they are still sharing a substantial amount of common genetic variability, while having completely different feeding habits. Moreover, sympatric species are exposed to the same environment in terms of chemical composition of the water, its thermal regime, etc., allowing less biased analysis of the influence of fish diet on composition and properties of gut bacterial communities. Additionally, such studies could help to shed light on the role of the gut microbiome in ecological niche segregation and speciation.

In the species complex *Coregonus lavaretus* there are found lacustrine sympatric pairs which serve as an intriguing model for studying different aspects of fish evolutionary biology (e.g.,  Ostbye et al., 2006; Rogers & Bernatchez, 2007; Lundsgaard-Hansen et al., 2013; Sevellec, Derome & Bernatchez, 2018). One among such sympatric whitefish pairs inhabits Teletskoye Lake in the Altai Mountains (West Siberia, Russia). This pair has characteristics common to other sympatric pairs of whitefish and segregated ecological niches (Bochkarev & Zuikova, 2006). *Coregonus lavaretus pidschian* (Gmelin, 1789) reaches 20–25 cm Smith’s fork length (at the age of seven years) and total body weight of around 150 g, while *C. l. pravdinellus* (Dulkeit, 1949) reaches only 17 cm Smith’s fork length (at the same age) and total body weight of around 40 g (Bochkarev, 2009). It has to be noted that *C. l. pidschian* is a long cycle whitefish with a maximum registered age of around 15 years while *C. l. pravdinellus* is a short cycle whitefish with a maximum registered age of around 7 years. Their origin, phylogenetic relationships and taxonomic position are still disputed. During the last 150 years *C. l. pravdinellus* and *C. l. pidschian* changed their taxonomical position several times being “natio”, “subspecies” and “species”. For instance, some authors consider *C. l. pidschian* as separate species *C. smitti* (Warpachowski, 1900; Politov, Gordon & Baldina, 2010). According to the analysis of mtDNA they are genetically very close to each other and share the same haplotypes (Bochkarev, Zuykova & Katokhin, 2011). Similar sympatric pairs are often referred to in the literature as normal (benthophages) and dwarf (planktophages) whitefish forms (e.g., Bernatchez, 2004). Due to the intricate taxonomical position of these fish and non-taxonomical aim of the present study, we will be considered these fish as a “forms”. These two forms will be hereafter referred to as “*pidschian*” and “*pravdinellus*”. It have been shown that *pravdinellus* has a narrow trophic specialization and feeds on zooplankton (different copepods and cladoceras) in both summer and winter periods whereas *pidschian* diet is more taxonomically diverse (larvae of insects, mollusks, gammarids, etc.) (Bochkarev & Zuikova, 2006). The diet of coregonids is correlated with their mouth position, and the number of gill rakers on the first branchial arch: *pidschian* has on average 28 rakers (few-rakered form) is benthivorous and has a downward facing mouth, whereas *pravdinellus* has on average 34 rakers (multi-rakered form) is planktivorous and the mouth faces anteriorly/semi-downward.

Despite considerable evolutionary and ecological research on such sympatric pairs of species within the *Coregonus lavaretus* complex, little is known about the relationships between the host diet and gut microbiome in such systems. Sevellec, Derome & Bernatchez (2018) have recently addressed the differences of the intestinal microbiota of “dwarf” and “normal” sympatric North-American whitefish pairs (*C. clupeaformis*) from different lakes and the bacterial community of the whitefish habitat water, as well as tested for possible parallelism in the microbiota of “dwarf” and “normal” whitefish. They used adherent bacterial DNA from an intestine segment isolated by rinsing the interior of the intestines with sterile saline solution. From our point of view, the major drawback of this approach when applied to fish with different feeding habits is that bacteria adhering by different mechanisms (e.g., receptor ligand junctions vs. biofilm production) and strength could be eliminated in different ratios and thus biasing further analysis. It is known that various bacteria inhabit different layers of the glycocalyx of the fish gut epithelium and possess different adherent ligands (Izvekova & Plotnikov, 2011), while the mucosa layer has species-specific biochemical and morphological features that may be influenced by different factors such as season of year, fish age and diet. Bacteria that inhabit the upper layer of the glycocalyx are the first line of host defense against pathogens by spatially limiting colonization by other species, or more actively by way of secretion of inhibitory compounds, making these bacteria extremely important for their host survival and physiology, but, if applying this approach, some of these bacteria could be rinsed out and eliminated from further analysis. Consequently, it will affect analyses of the bacterial composition and their predicted function. To avoid this, we adapted and applied a more comprehensive approach by subdividing the gut bacterial community into a number of distinctly defined groups: bacteria from the mucosal layer and bacteria from content for every part of the gut (Kashinskaya et al., 2017; Kashinskaya et al., 2018).

The main aim of the present study was to test for the composition and functional differences of gut bacterial communities of the sympatric forms *pidschian* and *pravdinellus*, and the microbiome environmental compartments from their surroundings using an in-depth approach by subdividing the gut tissue and its contents according to the defined morphological parts.

## Materials and Methods

### Study area and sampling

Fish were collected from the end of August to middle of September, 2017 in the north part of Lake Teletskoye (51.79°N; 87.30°E). Lake Teletskoye is a large (223 km^2^) and deep (325 m) oligotrophic lake (basin of Ob River) in the Altai Mountains (Altai Republic, Russia). The water temperature of the upper 1 m of the water column ranged from 17.1 to 18.3 °C, pH was 7.5–7.6. For comparative analysis of gastrointestinal microbiota we collected five individuals of *pidschian* (Smith’s fork length was 232.7 ± 5.8 mm) and five individuals of *pravdinellus* (Smith’s fork length was 144.3 ± 2.3 mm). All fish were captured using gill-nets (mesh sizes 18 and 22 mm) at depths from 2 to 15 m and transported alive to the laboratory in plastic containers with water from the lake (in approximately 30 min). All fish were sacrificed and treated as previously described (Kashinskaya et al., 2015). Gut was divided into six parts: cardiac stomach, pyloric stomach, pyloric caeca, anterior, middle, and posterior intestine. All studied individuals of *pidschian* and *pravdinellus* were checked for gut helminth infection and their total number was registered. Three out of five individuals of *pidschian* were infected by the cestode *Proteocephalus exiguus* (one tapeworm in every infected fish), whereas the five studied individuals of *pravdinellus* were not infected by helminthes in their gut.

For all parts of gut the mucosa and content samples were collected in aseptic condition. For the pyloric caeca only mucosa was collected. Due to small size of pyloric caeca, the mucosa was partially contaminated by content. Total DNA was extracted from 100 mg of each subsample. Some individuals had empty parts of stomach or intestine but corresponding mucosa samples were still used in further analysis. In addition, water, sediment, macrophyte (environmental microbiota) and invertebrates (microbiota of prey) samples were collected near to the shore where the fish nets were set. Water was sampled from the upper 0.5 m of the water column and pooled together from three locations in a sterile 3 l glass bottle. Water microbiota was collected by filtration of 500 ml of water onto 0.22 mm pore size polyethersulfone membrane filters (22 mm diameter, Millipore, EXPRESS PLUSTM, MerckKGaA, Germany). Sediment samples were collected in a total mass of 5 g using a Petersen grab. The samples of sediment from three locations were mixed and 100 mg was used to extract DNA. Scrapings from below water level of 2–3 trunks of three different macrophytes (*Butomus umbellatus*, *Potamogeton perfoliatus*, *Batrachium* sp.) were sampled with a spatula from an approximate depth of 0.3–0.5 m into sterile tubes. Approximate mass for DNA extraction was 100 mg of wet plant material. The choice of these macrophytes as environmental contributors to the fish gut microbiota was based on the dominance of these plants in the surrounding water body.

To better understand the environmental factors that influence the microbiota of the fish gut, 11 samples of invertebrates were also collected. Two integrated samples of zooplankton organisms (mainly copepods and cladocerans) were collected by filtering the water column from the bottom to the water surface using a Juday-type (125 µm mesh size) plankton net. Sample “Zooplankton 1” was collected near to the shore at one end of the gill-nets (at depth around 2 m) and the sample “Zooplankton 2” was collected near to the middle of the lake at the other end of the gill-nets (at depth around 15 m). The choice of invertebrates was based on the dominant taxa of food objects in fish gut content of *pidschian* and *pravdinellus* previously described by Bochkarev & Zuikova (2006). The microbiota from the whole body of the studied invertebrates was analysed. Before DNA extraction, the invertebrates were rinsed in sterile distilled water three times.

The present research has met the requirements guided by the order of the High and Middle Education Ministry (care for vertebrate animal included in scientific experiments, No 742 from 13.11.1984) and additionally by the Federal Law of the Russian Federation No 498 FL (from 19.12.2018) with regard to the humane treatment of animals.

### Sample preparation and DNA extraction

Before the DNA extraction, all 103 samples (86 from fish, six from environment, and 11 from invertebrates) were placed into sterile microcentrifuge tubes with lysis buffer for DNA isolation and mechanically homogenized by pestle for 1 min using a hand-held homogenizer. All samples were processed to extract DNA following the DNA-sorb B kit manufacturer’s protocols (kit for DNA extraction, Central Research Institute of Epidemiology, Russia).

### 16S rDNA library sequencing and processing

Genomic DNA extracted from the samples was amplified using primer pair (S-D-Bact-0341-b-S17 and S-D-Bact-0785-a-A-21) targeted to the V3–V4 region of the 16S rRNA genes (Klindworth et al., 2013). The forward (5′-TCGTCGGCAGCGTCAGATGTGTATAAGAGA-CAGCCTACGGGNGGCWGCAG-3′) and reverse (5′-GTCTCGTGGGCTCGGAGATGT-GTATAAGAGACAGGACTACHVGGGTATCTAATCC-3′) contained Illumina overhang adapter sequences (underlined regions). Amplicon libraries were subcontracted for preparation and sequencing using an Illumina MiSeq (500 cycles - 2 × 250 paired-end) by Evrogen (Moscow, Russia). Nucleotide sequences were deposited in the Sequence Read Archive (NCBI), accession number: PRJNA526436 and PRJNA526346. Sequencing failed for several samples of pyloric caeca thus these samples were excluded from further analysis.

Read pairs were merged and quality filtered with MOTHUR 1.31.2 (Schloss et al., 2009). Any reads with ambiguous sites and homopolymers of more than 8 bp were removed, as well as sequences shorter that 350 or greater than 500 bp. QIIME 1.9.1 (Caporaso et al., 2010) was used for further processing of the sequences. *De novo* (abundance based) chimera detection using USEARCH 6.1 (Edgar, 2010) was applied to identify possible chimeric sequences (‘identify_chimeric_seqs.py’ with an option ‘-musearch61’ in QIIME). After chimera filtering, the QIIME script ‘pick_open_reference_otus.py’ with default options was used to perform open-reference OTU picking by UCLAST (Edgar, 2010), taxonomy assignment (UCLAST, with a 0.80 confidence threshold), sequence alignment (PyNAST 1.2.2; Caporaso et al., 2010) and tree-building (FastTree 2.1.3; Price, Dehal & Arkin, 2010). This algorithm involves several steps of both closed-reference and open-reference OTU picking followed by taxonomy assignment, where the Greengenes core reference alignment (release ‘gg_13_8’; DeSantis et al., 2006) was used as a reference. Chloroplast, mitochondria and nonbacterial sequences were removed from further analysis.

### Analysis of alpha and beta diversity

The rarefaction curves for all samples are presented at Fig. S1. The richness (number of OTU’s and Chao1 index) and diversity estimates (Shannon and Simpson index) per sample were calculated using QIIME. Mann–Whitney pairwise test (*U* test) implemented in PAST 3.16 (Hammer, Harper & Ryan, 2001) was used to test for differences in alpha diversity measures between groups. The differences were considered significant at *P* ≤ 0.05.

The samples were then rarified to the lowest sequencing effort (479 sequences) and a weighted UniFrac dissimilarity matrix (Lozupone & Knight, 2005) was calculated in phyloseq R package (McMurdie & Holmes, 2013) and used for downstream analyses. Such sequencing effort allowed to include as many samples as possible in further analyses. Increasing the sequencing effort did not change the obtained results, but may reduce the number of analyzed samples. The matrix was used to perform principle coordinates analysis (PCoA) to visualize differences among groups of samples. To test the effect of various explanatory variables (whitefish forms, type of tissue [mucosa, content], part of gut, environmental compartments [prey, water, sediment and macrophytes]), on the groupings of bacterial communities, permutational multivariate analysis of variance using distance matrices were used as implemented in the ‘adonis’ function of the vegan R package (Oksanen et al., 2018). Pairwise comparisons for all pairs of levels of used factors were performed using the ‘adonis.pair’ function of EcolUtils R package (Salazar, 2018). Analysis of multivariate homogeneity of group dispersions (variances) to test if one or more groups is more variable than the others, was performed using the ‘betadisper’ function of the vegan R-package. In all the aforementioned tests statistical significance was determined by 10,000 permutations.

Venn diagrams of OTU membership were calculated using MetaCoMET (https://probes.pw.usda.gov/MetaCoMET/index.php). The membership method is the simplest and most standard method for determining the core microbiome using an OTU table, and is based on the presence or absence of OTUs among the different microbial communities being compared. If an OTU is observed at a level greater than the user-specified absolute or relative abundance threshold within any of the samples belonging to a specific group, it is counted as being a member of that group (the default value for the Membership parameter is 0).

### Functional analysis

The PICRUSt software package (Langille et al., 2013) was used to predict metagenome functional content of microbial communities. We generated the KEGG pathways (Kyoto encyclopedia of genes and genomes) and categorized functions to different gene categories at levels 1, 2, and 3. The categorized functions for different levels (frequency of occurrences of every group of genes in genome records) then were transformed to percentages from the total quantity of genes obtained and the differences between groups of samples were calculated by ANOSIM implemented in PAST (at *P* ≤ 0.05).

## Results

### Alpha diversity of the gut microbiota of fish and associated microbiota of environmental compartment

#### C. l. pidschian

The number of OTUs and Chao1 were significantly lower (*U* test, *P* ≤ 0.05) in mucosa than in the content from the corresponding parts of stomach and intestine. For the mucosa and content from both parts of the stomach, the average values of richness and diversity estimates were higher than from all parts of the intestine (for Chao1, OTU, and Shannon these differences were significant, *U* test, *P* ≤ 0.05). In all cases the number of OTUs was significantly higher (U test, *P* ≤ 0.05) in gut content than in the corresponding part of the gut mucosa (Table 1; Table S1).

**Table 1:** Diversity analysis of microbial community of fish gut, their prey and environmental compartments in Teletskoye Lake.

Type of sample				Number of samples	Richness estimates		Diversity estimates	
					Number of observed OTU’s	Chao1	Shannon	Simpson
*Fish*								
*C. l. pidschian*	Mucosa	Stomach	Cardiac	5	755 ± 257.5	897 ± 257.6	6.68 ± 0.60	0.94 ± 0.03
			Pyloric	5	1,068 ± 130.2	1,374 ± 157.1	5.43 ± 1.21	0.74 ± 0.13
				**Mean ± SE**	**911.1 ± 145.7**	**1,135.5 ± 163.0**	**6.06 ± 0.67**	**0.84 ± 0.07**
		Intestine	Pyloric caeca	2	87 ± 28.5	135 ± 44.8	4.43 ± 0.52	0.92 ± 0.03
			Anterior	5	215 ± 35.5	365 ± 89.3	4.39 ± 0.85	0.78 ± 0.11
			Middle	5	150 ± 18.7	251 ± 39.6	3.59 ± 0.66	0.76 ± 0.09
			Posterior	4	109 ± 6.6	205 ± 11.3	2.78 ± 0.95	0.57 ± 0.18
				**Mean ± SE**	**152.1 ± 17.1**	**260.8 ± 35.1**	**3.74 ± 0.42**	**0.74 ± 0.06**
	Content	Stomach	Cardiac	3	1,678 ± 356.7	2,114 ± 441.8	5.27 ± 1.10	0.77 ± 0.13
			Pyloric	5	1,861 ± 300.7	2,372 ± 322.6	7.66 ± 0.54	0.95 ± 0.04
				**Mean ± SE**	**1,792.3 ± 216.9**	**2,275.3 ± 245.6**	**6.76 ± 0.65**	**0.88 ± 0.06**
		Intestine	Anterior	5	895 ± 184.5	1,221 ± 236.2	4.44 ± 1.14	0.72 ± 0.11
			Middle	3	823 ± 297.7	1,135 ± 410.7	4.64 ± 1.41	0.78 ± 0.14
			Posterior	3	793 ± 199.3	1,118 ± 285.0	5.51 ± 1.05	0.89 ± 0.06
				**Mean ± SE**	**847.5 ± 115.7**	**1,169.2 ± 154.9**	**4.79 ± 0.65**	**0.78 ± 0.06**
*C. l. pravdinellus*	Mucosa	Stomach	Cardiac	5	190 ± 61.3	304 ± 99.8	4.17 ± 0.55	0.84 ± 0.07
			Pyloric	5	195 ± 44.2	317 ± 41.1	4.53 ± 0.44	0.87 ± 0.04
				**Mean ± SE**	**192.4 ± 35.6**	**310.2 ± 50.9**	**4.35 ± 0.34**	**0.86 ± 0.04**
		Intestine	Pyloric caeca	3	132 ± 27.0	321 ± 77.2	2.33 ± 1.08	0.59 ± 0.17
			Anterior	5	152 ± 48.7	442 ± 81.3	2.25 ± 0.47	0.59 ± 0.09
			Middle	5	119 ± 27.2	240 ± 56.2	2.73 ± 0.35	0.70 ± 0.04
			Posterior	4	74 ± 15.2	139 ± 33.2	2.69 ± 0.57	0.64 ± 0.12
				**Mean ± SE**	**120.4 ± 17.5**	**260.6 ± 35.8**	**2.51 ± 0.26**	**0.63 ± 0.05**
	Content	Stomach	Cardiac	2	590 ± 28.5	737 ± 44.5	5.20 ± 0.04	0.90 ± 0.01
			Pyloric	3	408 ± 72.0	544 ± 78.1	4.36 ± 0.68	0.83 ± 0.06
				**Mean ± SE**	**480.6 ± 60.1**	**621.2 ± 65.3**	**4.70 ± 0.43**	**0.85 ± 0.04**
		Intestine	Anterior	3	293 ± 97.2	503 ± 177.3	1.50 ± 0.46	0.39 ± 0.11
			Middle	3	229 ± 100.2	428 ± 186.8	2.82 ± 1.06	0.67 ± 0.17
			Posterior	3	190 ± 43.9	378 ± 110.5	2.34 ± 0.26	0.62 ± 0.10
				**Mean ± SE**	**237.2 ± 44.9**	**436.6 ± 82.9**	**2.22 ± 0.39**	**0.56 ± 0.08**
*Prey*								
Gammaridae sp.			Whole	1	564	709	5.91	0.96
Hirudinea sp.			Whole	1	922	1,179	4.91	0.92
*Lymnaea* sp.			Whole	1	2,178	2,823	8.53	0.99
Notonectidae sp.			Whole	1	829	1,035	5.14	0.93
*Planorbis* sp.			Whole	2	2,050 ± 58.5	2,574 ± 113.1	7.37 ± 0.35	0.97 ± 0.01
Trichoptera sp. (larvae with protective case)			Whole	2	5,637 ± 881.0	7,102 ± 552.4	9.92 ± 0.09	1.00 ± 0.00
Trichoptera sp. (larvae without protective case)			Whole	1	5,398	6,918	10.08	1.00
Zooplanktons			Whole	2	3,349 ± 172.0	4,112 ± 151.4	7.19 ± 0.42	0.96 ± 0.02
				**Mean ± SE**	**2,905.6 ± 594.4**	**3,658.2 ± 739.9**	**7.59 ± 0.56**	**0.97 ± 0.01**
*Environmental compartments*								
Water			500 ml	1	2,588	3,189	6.78	0.97
Sediment			0.1 g	1	6,154	7,101	9.38	0.98
*Butomus umbellatus*			Scrapings 0.1 g	1	648	830	1.64	0.46
*Potamogeton perfoliatus*			Scrapings 0.1 g	1	801	1,047	2.54	0.47
*Batrachium* sp.			Scrapings 0.1 g	1	2,834	3,543	7.50	0.98
Epiphytes			Scrapings 0.1 g	1	1,455	1,734	8.04	0.98
				**Mean ± SE**	**2413.3 ± 833.4**	**2907.3 ± 952.7**	**5.98 ± 1.28**	**0.81 ± 0.11**

The number of OTU and Chao1 index were significantly higher in mucosa from pyloric stomach (1,068 ± 130.2 and 1,374 ± 157.1 respectively) than in mucosa from cardiac stomach (755 ± 257.5 and 897 ± 257.6 respectively). At the same time, for content from the same parts of stomach significant differences (*U* test, *P* ≤ 0.05) were observed only for the Shannon index (Table 1; Table S1).

The OTU number and Chao1 in mucosa from cardiac and pyloric stomach were significantly lower (*U* test, *P* ≤ 0.05) than in prey objects whereas for content from the same parts of stomach these differences were insignificant. For different parts of mucosa from the intestine the richness and diversity estimates were significantly different (*U* test, *P* ≤ 0.05) if compared with these estimates from the prey. Similar relationships were found between content from different parts of intestine and prey. Significant differences (U test, *P* ≤ 0.05) for OTU number and Chao1 were found between mucosa from different parts of the intestine and environmental compartments (water, sediments, and plants), whereas neither stomach mucosa and content nor intestinal content had significant differences in terms of richness and diversity estimates (Table 1; Table S1).

#### C. l. pravdinellus

Significant differences among mucosa from different parts of intestine were observed for richness estimates (Chao1 and OTU) whereas for diversity estimates there were no significant differences. At the same time, the diversity estimates were significantly higher in mucosa and content of both parts of stomach than in mucosa and content from all parts of intestine (Table 1; Table S1).

There were significant differences (U test, *P* ≤ 0.05) between content from cardiac and pyloric stomach in terms of richness and diversity estimates. There were significant differences between content from both parts of stomach and all parts of intestine for OTU, Shannon, and Simpson indices (U test, *P* ≤ 0.05). The OTU number and Chao1 index were significantly higher (U test, *P* ≤ 0.05) in mucosa from cardiac and pyloric stomach than the content from these parts, whereas for intestine the significant differences were found only in mucosa of the posterior intestine and its content. In all cases the number of OTUs was significantly higher (U test, *P* ≤ 0.05) in content than in the corresponding part of the gut mucosa (Table 1; Table S1).

The richness and diversity estimates were significantly different between mucosa and content from different parts of the stomach and intestine if compared with the same estimates from prey. In terms of the number of OTUs and Chao1 estimates there were differences found between mucosa and content from all parts of the gut (Table 1; Table S1).

#### Between forms

The number of OTUs and Chao1 index in mucosa and content of cardiac and pyloric stomach were significantly different between *pidschian* and *pravdinellus*. The significant differences were observed between *pidschian* and *pravdinellus* for content from different parts of intestine in terms of OTU number and Chao1 indices whereas for mucosa from the same parts of intestine these differences were absent. No significant differences were found for diversity estimates of mucosa and content of different parts of the gut (there were a few exceptions) between *pidschian* and *pravdinellus* (Table 1; Table S1).

### Microbiota composition of gut mucosa and content of fish

#### C. l. pidschian

There were found significant effects on the composition of the microbiota from each part of the gut (ADONIS, *p* < 0.001, *R*^2^ = 0.24) and sample type (mucosa or content) (ADONIS, *p* = 0.04, *R*^2^ = 0.04) (Tables S3,  S4,  S5A). No significant differences were obtained between mucosa of cardiac and pyloric stomach (ADONIS, *p* = 0.36, *R*^2^ = 0.13) (Table S3), nor between content from cardiac and pyloric stomachs (ADONIS, *p* = 0.28, *R*^2^ = 0.19) (Table  S4). No significant differences were found for mucosa and content among pyloric caeca, anterior, middle and posterior intestine (ADONIS, *p* ≥ 0.05) (Tables S4,  S6B). If it is taken into account that the effect of sample type is negligible and unite mucosa and content together, then, the differences between anterior and posterior intestine becomes significant (ADONIS, *p* = 0.016, *R*^2^ = 0.15) (Table S5B). Beta diversity for each part of the gut (mucosa and content inclusive) is presented in Fig. S2.

**Figure 1:**
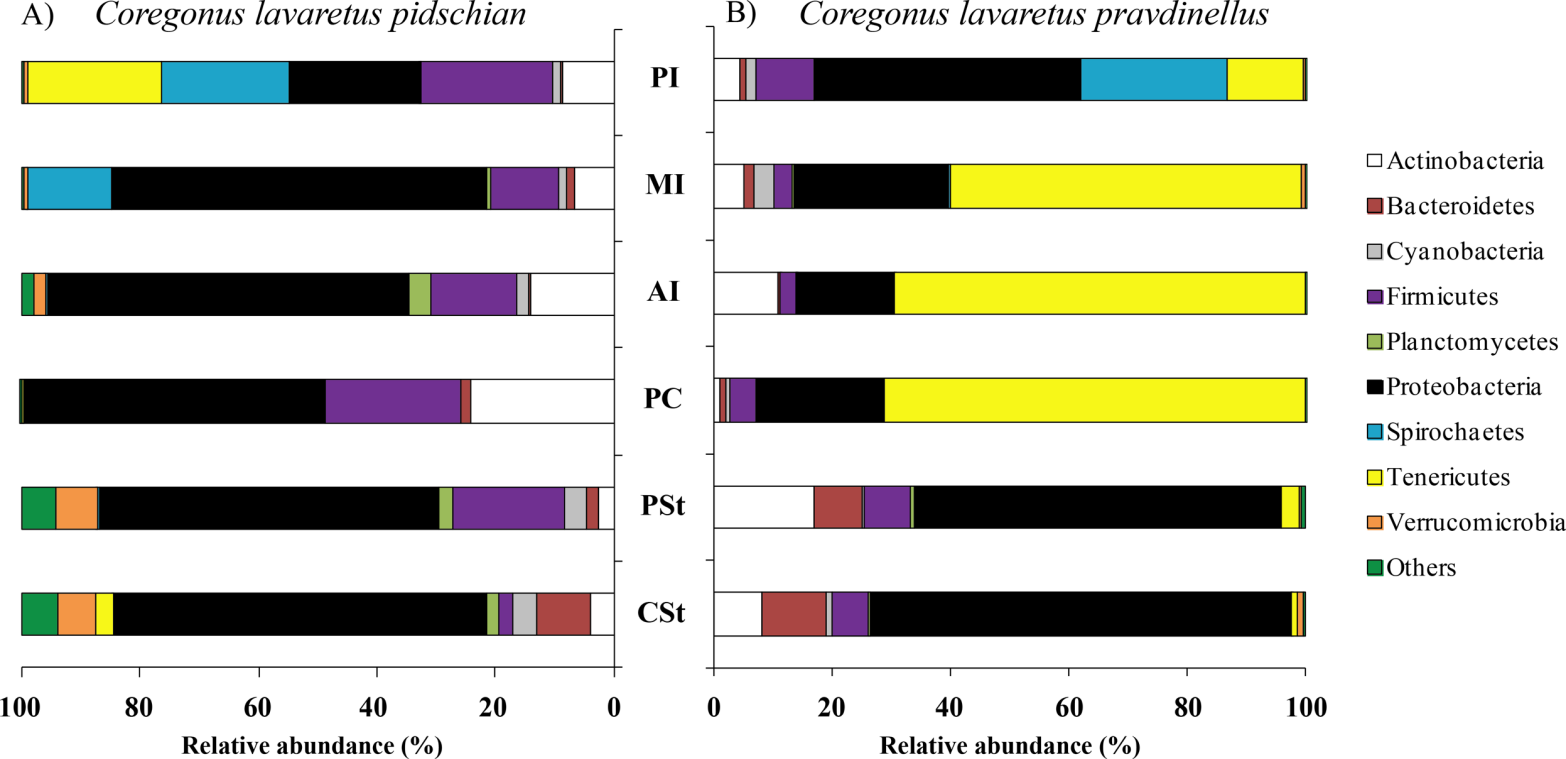
Phylum composition of microbiota from gut mucosa of *C. l. pidschian* (A) and *C. l. pravdinellus* (B). CSt, cardiac stomach; PSt, pyloric stomach; PC, pyloric caeca; AI, anterior intestine; MI, middle intestine; PI, posterior intestine.

**Figure 2:**
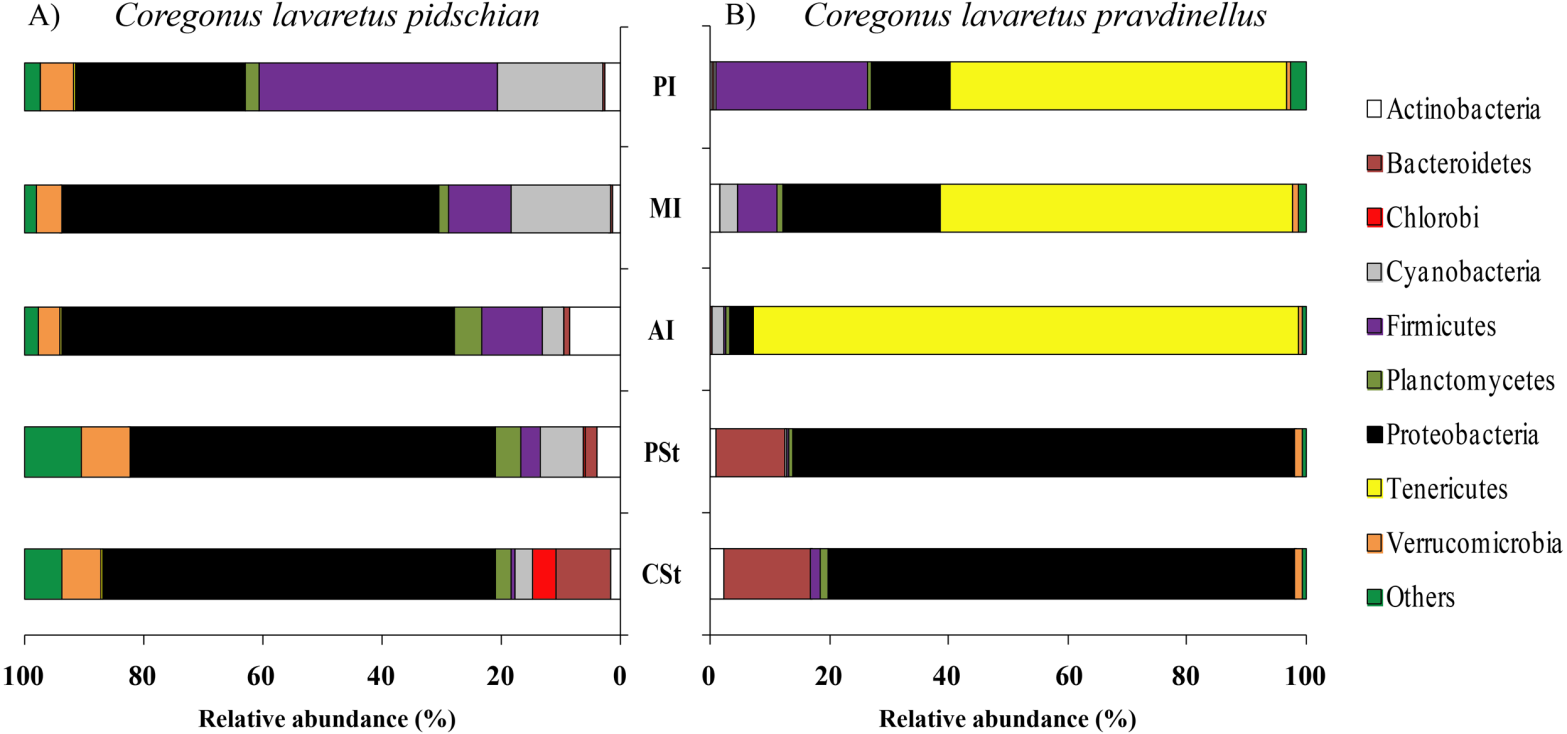
Phylum composition of microbiota from gut content of *C. l. pidschian* (A) and *C. l. pravdinellus* (B). CSt, cardiac stomach; PSt, pyloric stomach; AI, anterior intestine; MI, middle intestine; PI, posterior intestine.

Sixty and twenty phyla were registered in the mucosa of cardiac and pyloric stomach, respectively. Proteobacteria was the most dominant phyla in mucosa and content of cardiac (63.1 ± 2.3% and 66.2 ± 10.3%, respectively) and pyloric stomach (57.3 ± 13.8% and 66.4 ± 2.1%, respectively) (Figs. 1 and 2).

Eight, fourteen, fifteen, and fifteen phyla were registered in the mucosa of pyloric caeca, anterior, middle, and posterior intestine, respectively. In all parts of intestine (pyloric caeca, anterior, middle, and posterior) the dominant phyla in mucosa were Proteobacteria (38.8 ± 7.5%) and Firmicutes (15.6 ± 3.9%). In the mucosa of the posterior part of the intestine the phylum Spirochaetes was found in significant amount 21.6 ± 21.0% (Fig. 1). Twenty-two, eighteen, and eighteen phyla were registered in the content from the anterior, middle, and posterior intestine, respectively. In the content obtained from anterior, middle and posterior intestines the dominant bacterial phyla were the same as were described for mucosa from the same parts of the intestine. The phylum Spirochaetae was almost absent in the intestinal content 0.16 ± 0.15% (Fig. 2).

**Figure 3:**
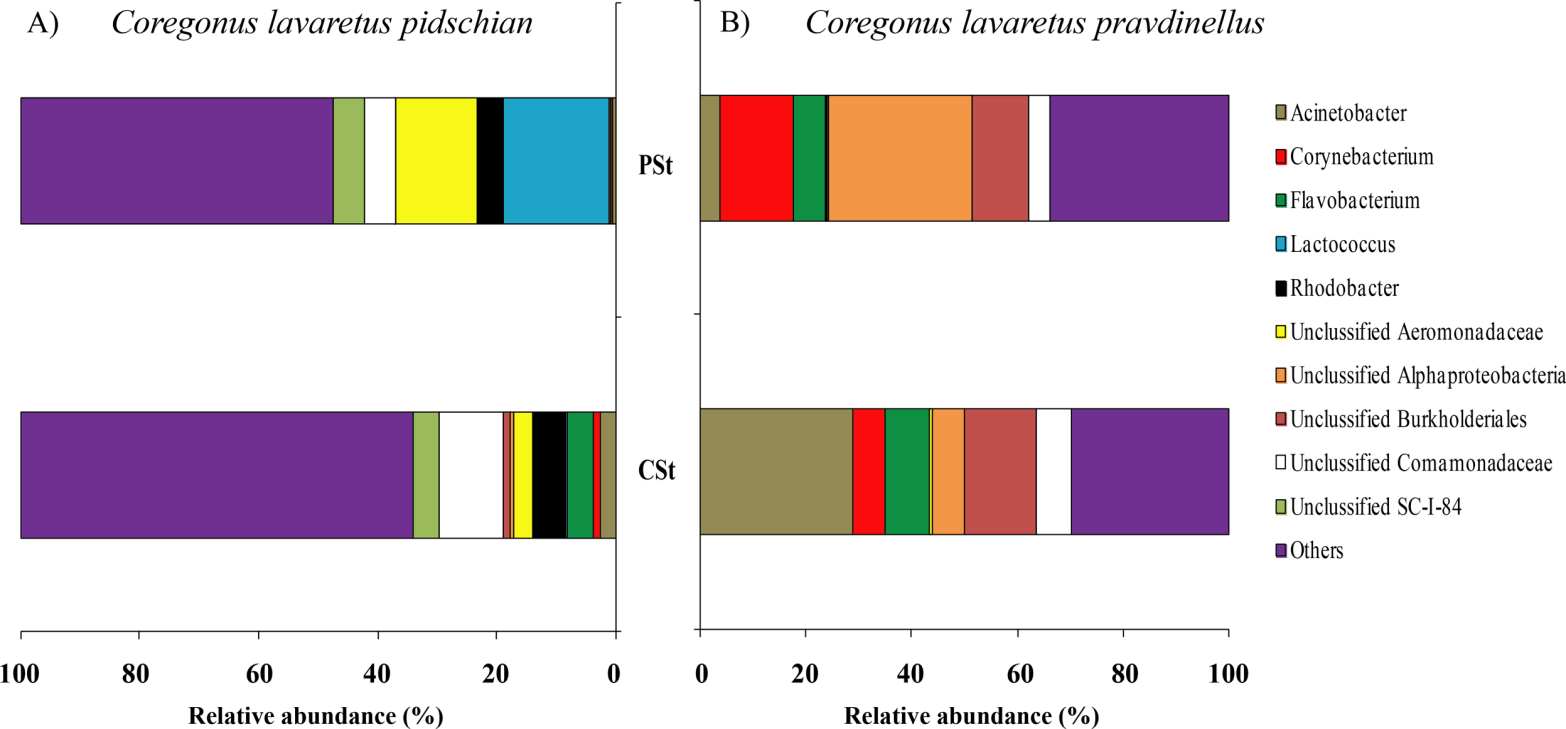
Composition of microbiota at the genus level in stomach mucosa of *C. l. pidschian* (A) and *C. l. pravdinellus* (B). CSt, cardiac stomach; PSt, pyloric stomach.

According to the dominant group of bacteria at the genus level with highest levels of prevalence, the mucosa and content of stomach and intestine were different from each other. In the stomach mucosa the dominant groups were *Lactococcus* (9.1 ± 8.8%), Unclassified Aeromonadaceae (8.4 ± 6.4%), and Unclassified Comamonadaceae (7.9 ± 1.6%) (Fig. 3).

**Figure 4:**
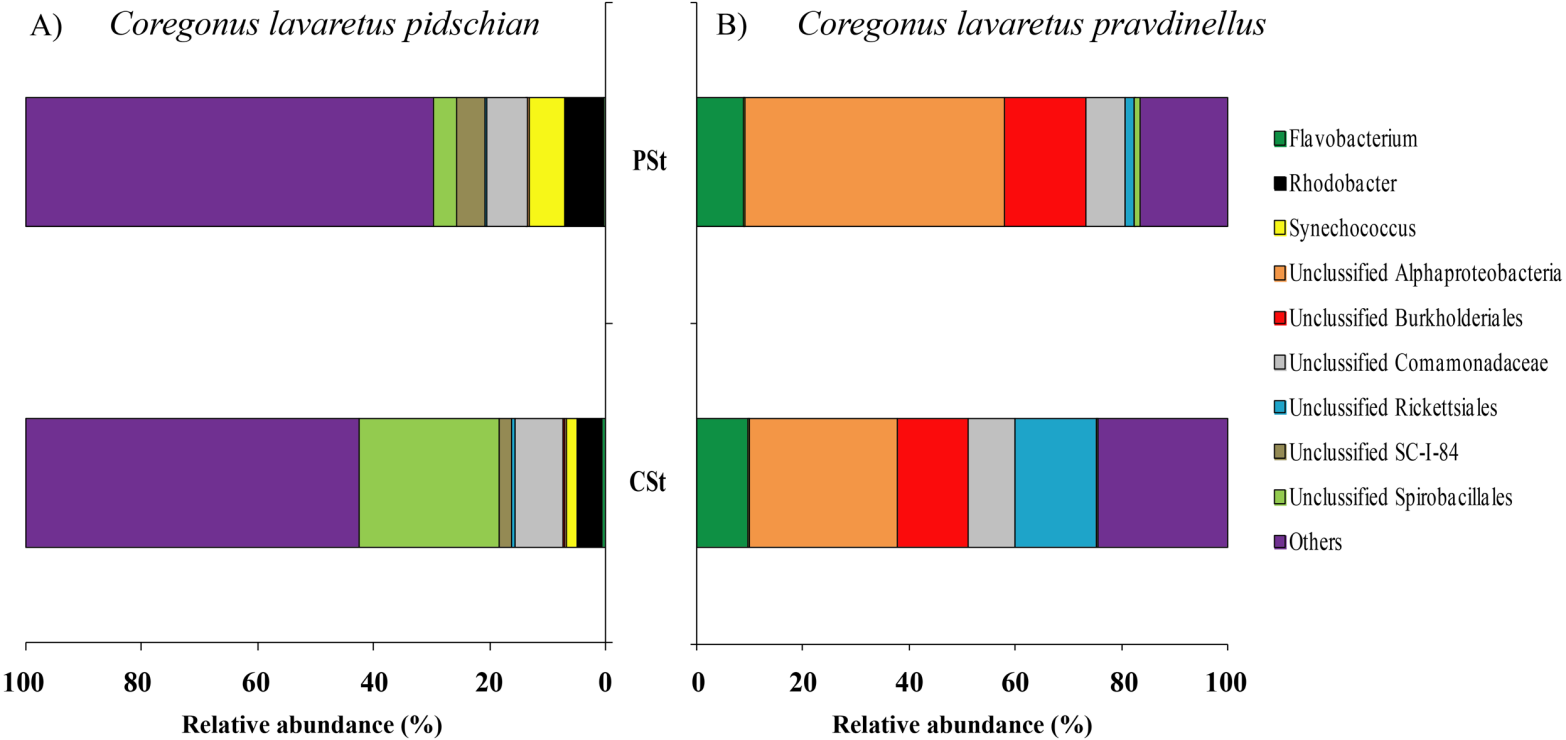
Composition of microbiota at the genus level in stomach content of *C. l. pidschian* (A) and *C. l. pravdinellus* (B). CSt, cardiac stomach; PSt, pyloric stomach.

In the stomach content the highest level of prevalence the dominant bacterial groups were represented by Unclassified Spirobacillales (11.6 ± 5.1%) and Unclassified Comamonadaceae (7.4 ± 1.7%) (Fig. 4).

**Figure 5:**
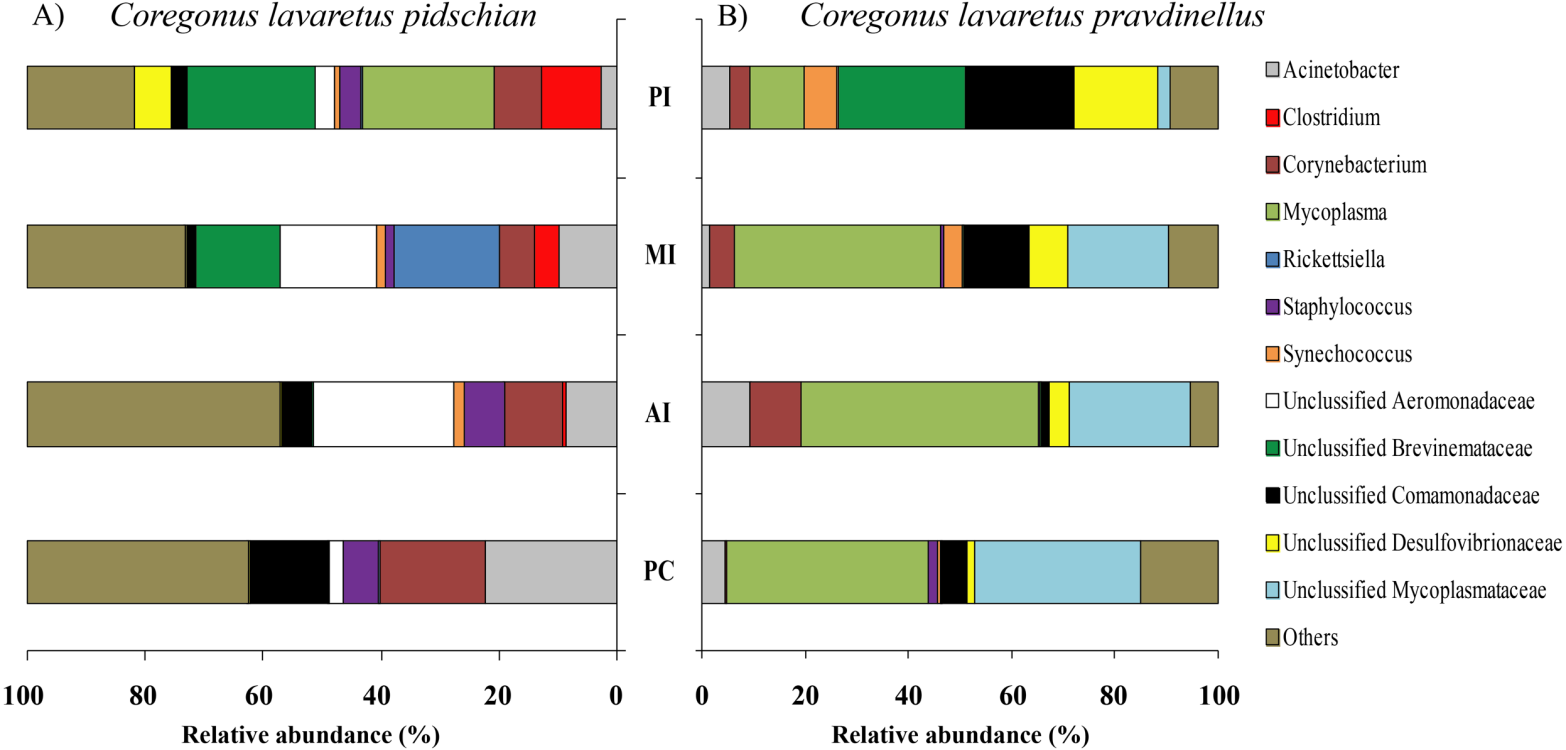
Composition of microbiota at the genus level in intestinal mucosa of *C. l. pidschian* (A) and *C. l. pravdinellus* (B). PC, pyloric caeca; AI, anterior intestine; MI, middle intestine; PI, posterior intestine.

In mucosa of the pyloric caeca at the genus level the dominant bacterial taxa were presented by *Acinetobacter* (22.3 ± 8.9%), *Corynebacterium* (17.7 ± 10.2%) and Unclassified Comamonadaceae (13.5 ± 4.3%) (Fig. 5); in mucosa of the anterior intestine –Unclassified Aeromonadaceae (23.9 ± 16.1%); in mucosa of the middle intestine –Rickettsiella (18.0 ± 18.0%), Unclassified Aeromonadaceae (16.3 ± 5.9%), Unclassified Brevinemataceae (14.4 ± 14.4%); in mucosa of the posterior intestine –*Mycoplasma* (22.5 ± 22.3%) and Unclassified Brevinemataceae (21.6 ± 21.0%) (Fig. 5).

**Figure 6:**
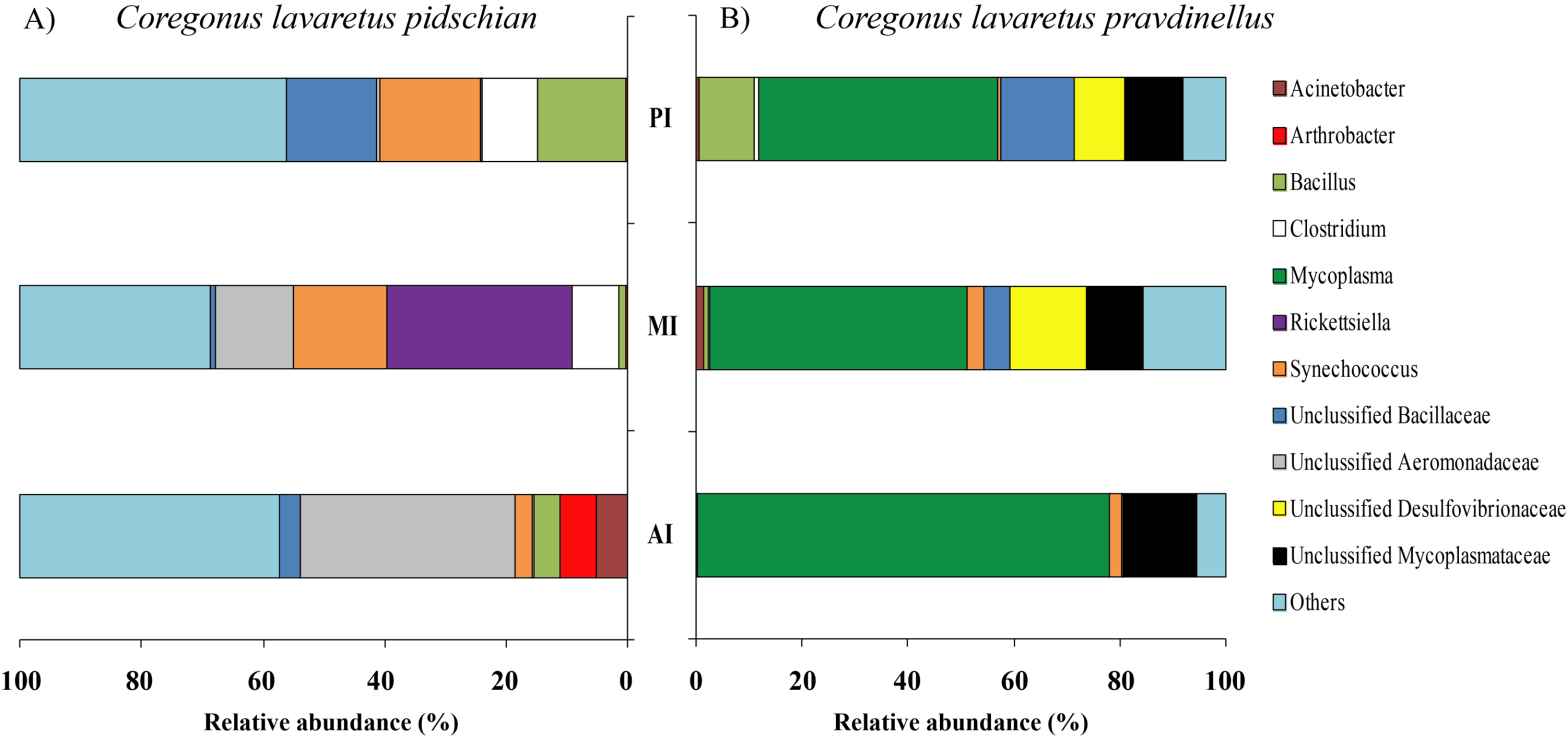
Composition of microbiota at the genus level in intestinal content of *C. l. pidschian* (A) and *C. l. pravdinellus* (B). AI, anterior intestine; MI, middle intestine; PI, posterior intestine.

The dominant group of bacteria at the genus level from content of anterior intestine was represented by only Unclassified Aeromonadaceae with prevalence 35.4 ± 19.3% (Fig. 6). In the content from middle intestine the dominant bacterial group was represented by Rickettsiella (30.7 ± 30.7%), *Synechococcus* (15.4 ± 9.9%), and Unclassified Aeromonadaceae (12.7 ± 10.9%). In the posterior intestine three dominant groups with equal level of prevalence were detected (*Synechococcus*—16.4 ± 6.7%, Unclassified Bacillaceae—14.9 ± 9.1%, and *Bacillus*—14.6 ± 14.1%) (Fig. 6).

According to Venn diagrams of OTU membership, the core microbiota among mucosa and content of stomach and intestine was represented by 399 OTUs (Fig. S4). In terms of per sent it was 5.8 and 9.9% from content of stomach and intestine, and 22.6 and 33.2% from mucosa of stomach and intestine respectively.

#### C. l. pravdinellus

The segment of the gut examined had a significant determinative effect on the composition of the microbiota (ADONIS, *p* < 0.05, *R*^2^ = 0.50) (Tables S3,  S4,  S5A). No significant differences were obtained between mucosa of cardiac and pyloric stomach for *pravdinellus* (ADONIS, *p* = 0.43, *R*^2^ = 0.12) (Table S3). The differences between content from cardiac and pyloric stomachs were insignificant as well (ADONIS, *p* = 0.44, *R*^2^ = 0.25) (Table S4). No significant differences were found for mucosa and content between pyloric caeca, anterior, middle and posterior intestine (ADONIS, *p* ≥ 0.05) (Tables S4,  S6A). If it is taken into account that the effect of sample type (mucoas vs. content) is negligible and unite mucosa and content together, then, the differences between anterior and posterior intestine will be significant (ADONIS, *p* = 0.039, *R*^2^ = 0.18) (Table S5B). Beta diversity according to segment of gut from *pravdinellus* (mucosa and content inclusive) is presented in Fig. S2.

Thirty and thirty-four phyla were registered in the mucosa of cardiac and pyloric stomach, respectively. Proteobacteria was the most dominant phyla in mucosa and content of cardiac (71.2 ± 11.9% and 78.3 ± 1.3%, respectively) and pyloric stomach (62.3 ± 12.4% and 84.0 ± 5.9%, respectively) (Figs. 1 and 2).

Eleven, thirteen, thirteen, and eleven phyla were registered in the mucosa of pyloric caeca, anterior, middle, and posterior intestine, respectively. In all parts of intestinal mucosa (pyloric caeca, anterior, middle, and posterior) the dominant phyla were Tenericutes (49.6 ± 9.9%) and Proteobacteria (28.1 ± 6.7%) (Fig. 1). In the mucosa of the posterior part of intestine of the phylum Spirochaetes was found in significant amount 24.9 ± 18.5% (Fig. 1). Thirteen, twelve, and fourteen phyla were registered in the content from anterior, middle, and posterior intestine, respectively. In the content obtained from anterior, middle and posterior intestines the dominant bacterial phyla were the same that were described for mucosa from the same parts of the intestine. The phylum Spirochaetes was almost absent in intestinal content 0.86 ± 0.82% (Fig. 2).

According to the dominant group of bacteria at the lowest taxonomical level with highest level of prevalence, the mucosa and content of stomach and intestine were different from each other. In stomach mucosa the dominant groups were Unclassified Alphaproteobacteria (16.6 ± 7.0%), *Acinetobacter* (16.2 ± 9.5%), and Unclassified Burkholderiales (12.1 ± 5.1%) (Fig. 3). In stomach content the dominant bacterial groups were represented by Unclassified Alphaproteobacteria (40.5 ± 7.7%), Unclassified Burkholderiales (14.4 ± 2.9%) (Fig. 4).

In mucosa of pyloric caeca, anterior, and middle intestine the dominant groups of bacteria were represented by *Mycoplasma* (39.0 ± 17.9, 45.8 ± 14.3, and 40 ± 8.1%, respectively) and Unclassified Mycoplasmataceae (32.1 ± 19.6, 23.5 ± 11.5, and 19.3 ± 9.0%, respectively) (Fig. 5). In the posterior intestine the dominant group of bacteria was represented by Unclassified Brevinemataceae (24.9 ± 18.5%), Unclassified Comamonadaceae (21 ± 18.4%), Unclassified Desulfovibrionaceae (16.5 ± 16.0%), and *Mycoplasma* (10.4 ± 4.3%) (Fig. 5).

The dominant group of bacteria at the lowest taxonomical level from content of anterior intestine was represented by *Mycoplasma* and Unclassified Mycoplasmataceae with prevalence 77.9 ± 7.6% and 13.6 ± 4.0%, respectively (Fig. 6). The dominant bacterial group in the content from middle intestine was presented by *Mycoplasma* (48.4 ± 17.1%), Unclassified Desulfovibrionaceae (14.5 ± 14.0%), and Unclassified Mycoplasmataceae (10.7 ± 4.0%). In the posterior intestine the dominant bacterial group was represented by *Mycoplasma* (45.2 ± 20.5%) and three other groups with similar level of the prevalence (10–13%): Unclassified Bacillaceae, Unclassified Mycoplasmataceae and *Bacillus* (Fig. 6).

According to Venn diagrams of OTU membership, the core microbiota among mucosa and content of stomach and intestine was represented by 153 OTUs (Fig. S4). In terms of percentage it was 12.1 and 13.7% from content of stomach and intestine, and 15.1 and 17.3% from mucosa of stomach and intestine respectively.

#### Between forms

The *Coregonus* forms of whitefish and the segment of the digestive system were factors with a significant determinative effect on the microbiota from gut mucus and content (Table S6A). The differences were significant for mucosa of pyloric stomach between *pidschian* and *pravdinellus* (ADONIS, *p* = 0.035, *R*^2^ = 0.30) whereas for mucosa of cardiac stomach the differences were insignificant (ADONIS, *p* = 0.08, *R*^2^ = 0.21). The differences were insignificant for content of cardiac (ADONIS, *p* = 0.125) and pyloric (ADONIS, *p* = 0.088) parts of the stomach between *pidschian* and *pravdinellus* (Table S7). Beta diversity by whitefish *Coregonus* forms and gut segment for mucosa and content is presented in Fig. S2.

The differences were significant between *pidschian* and *pravdinellus* for mucosa of anterior (ADONIS, *p* = 0.043, *R*^2^ = 0.47) and middle (ADONIS, *p* = 0.035, *R*^2^ = 0.50) intestine (Table S5B) whereas for content from the same parts of intestine the differences were insignificant (ADONIS, *p* ≥ 0.05) (Table S7). The bacterial community of the environment was significantly different from bacterial communities of all parts of gut mucosa (ADONIS, *p* ≥ 0.05) except mucosa of the cardiac stomach for *pidschian* and *pravdinellus* (Fig. S3). The bacterial community of gut content of *pidschian* and *pravdinellus* was significantly different from the environment in almost all cases (ADONIS, *p* < 0.05) (Table S4). The bacterial community of prey was significantly different from bacterial communities of all parts of the gut, both content and mucosa (ADONIS, *p* < 0.05), except for the content and mucosa of the cardiac stomach of *pidschian* (Tables S3,  S4). The Beta diversity by species and segment of the gut for mucosa and content is presented in Fig. S2.

The differences were significant for mucosa of cardiac and pyloric stomach in the quantity of Verrucomicrobia, Planctomycetes, Acidobacteria, and Chloroflexi (U test, *p* < 0.05). When comparing content from pyloric stomach for *pidschian* and *pravdinellus*, the differences were significant (U test, *p* < 0.05) for all bacterial phyla with the exception of Planctomycetes and Nitrospirae, whereas for content from cardiac stomach no differences were found (U test, *p* < 0.05).

The differences between each species were significant for mucosa of the anterior intestine in the quantity of Proteobacteria, Firmicutes, Planctomycetes, Verrucomicrobia, and Tenericutes (U test, *p* < 0.05). Also, differences were significant for mucosa of the middle intestine in regard to the quantity of Firmicutes and Tenericutes (U test, *p* < 0.05). For content from anterior intestine the differences between *pidschian* and *pravdinellus* were significant (U test, *p* < 0.05) for Proteobacteria and Tenericutes (U test, *p* < 0.05).

According to the dominant groups of bacteria at the genus level with highest level of prevalence, the mucosa and content of stomach and intestine were different *pidschian* and *pravdinellus*.

According to Venn diagrams of OTU membership, the core microbiota between *pidschian* and *pravdinellus* for stomach mucosa and content was represented by 223 OTUs (Fig. S4). In terms of percentage it was 3.2 and 4.4% from content and mucosa of the stomach of *pidschian* and 17.6 and 22.0% from content and mucosa of stomach of *pravdinellus* respectively. The core microbiota between *pidschian* and *pravdinellus* for intestinal mucosa and content was represented by 102 OTUs (Fig. S4). In terms of percentage it was 2.5 and 8.5% from content and mucosa of intestinal of *pidschian* and 9.1 and 11.5% from content and mucosa of intestinal of *pravdinellus* respectively.

### Microbiota associated with prey of fish and environmental compartments

**Figure 7:**
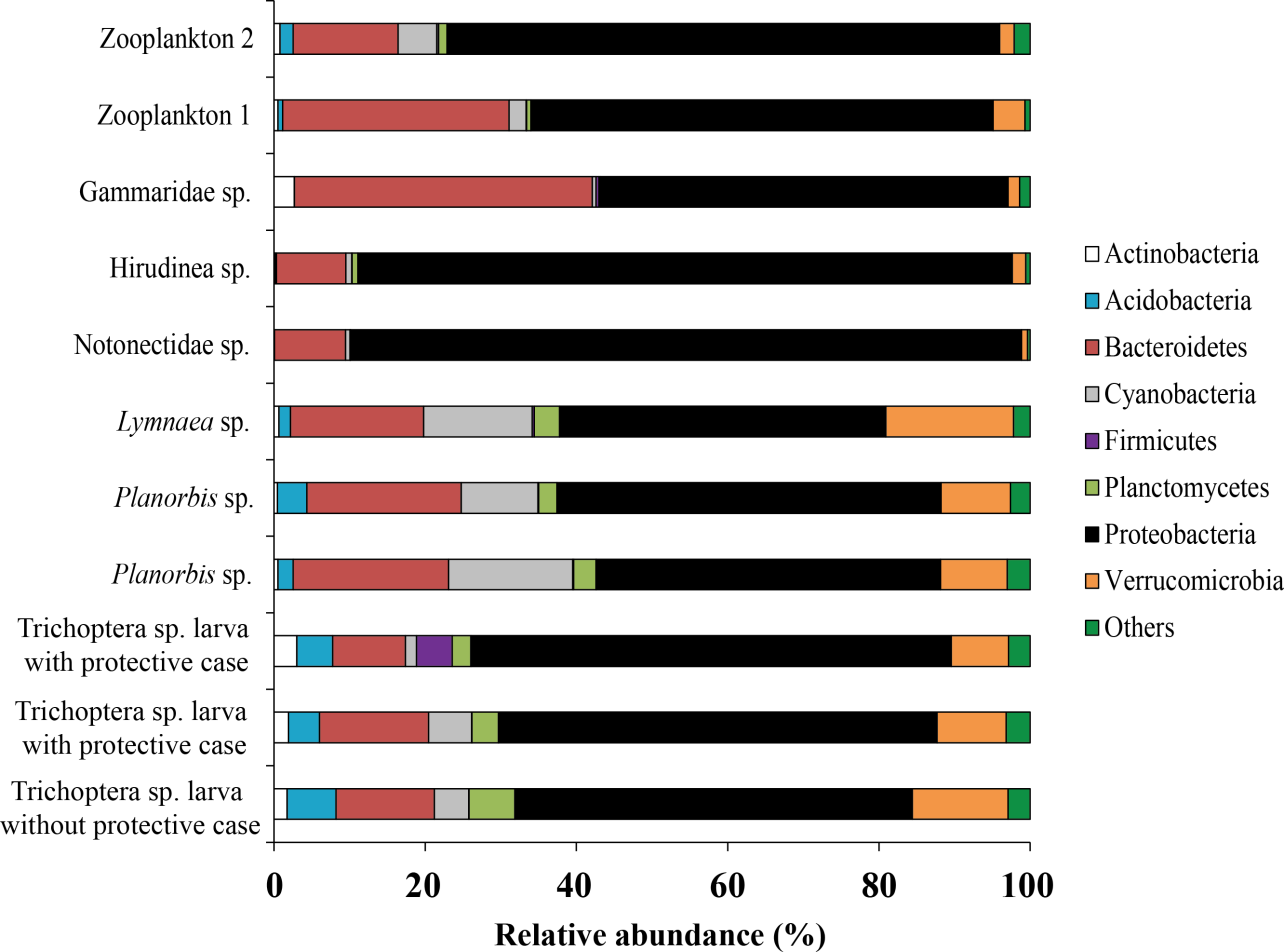
The associated microbiota of fish prey at phylum level in Teletskoye Lake.

Forty five bacterial phyla were identified from the associated microbiota of prey (aquatic invertebrates) (Fig. 7). From each prey, the phylum Proteobacteria made up the majority of all sequences although varying among different prey from 43.1 to 88.7% (mean is 61.5 ± 4.6%). Bacteroidetes was the second most common phylum, varying in abundance from 9.2 to 39.4% (mean is 17.9 ± 2.8%) among prey.

**Figure 8:**
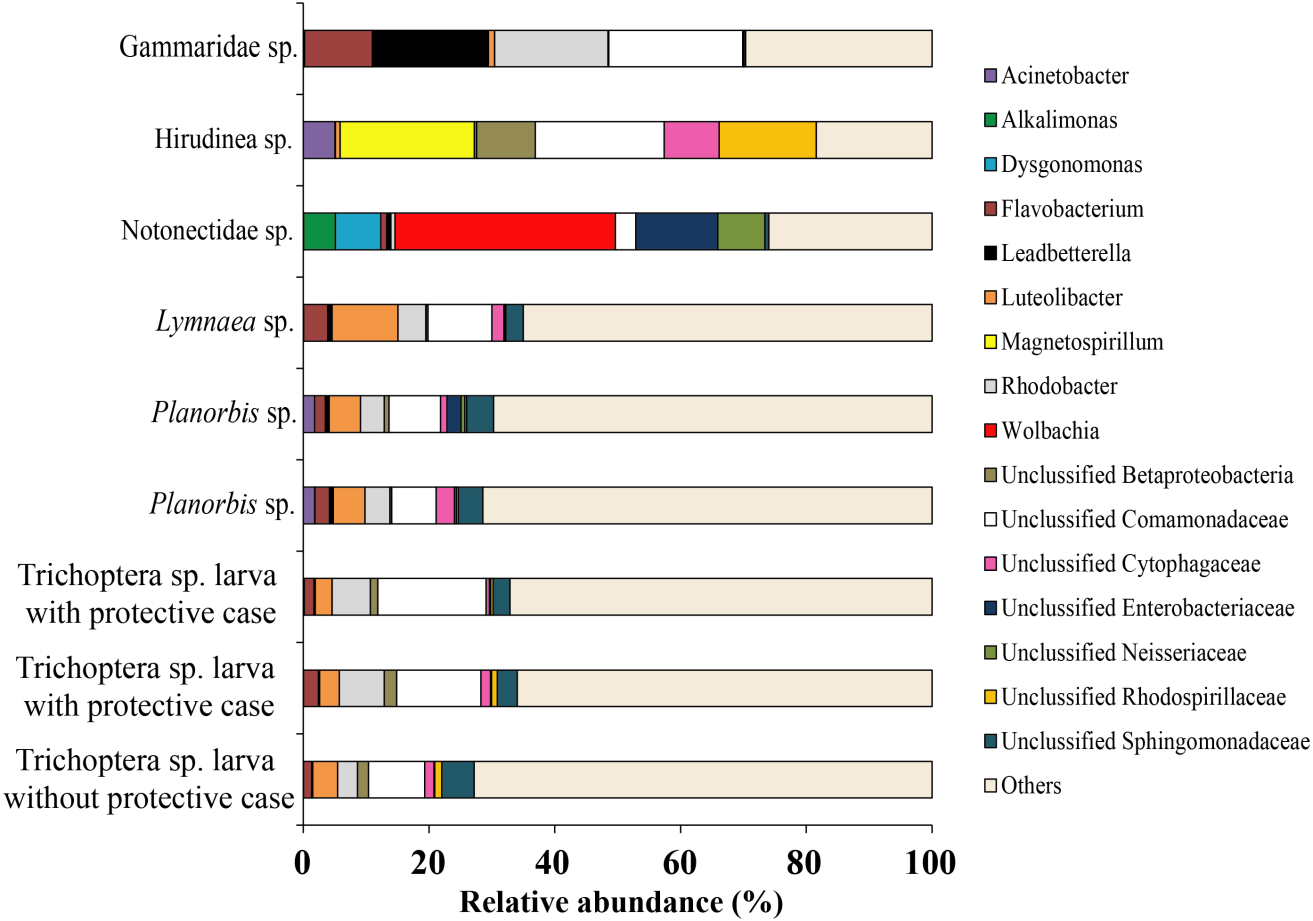
The associated microbiota of fish prey (without zooplanktons) at the genus level in Teletskoye Lake.

Among all studied invertebrates (backswimmer was exception) the dominant microbiota among observed OTU’s at the genus level was represented by Unclassified Comamonadaceae with variation from 7.1 ± 0.5% (*Planorbis* sp.) to 21.4 (Gammaridae sp.). The second most common genera, associated with caddis fly larva and mollusks were *Rhodobacter* (5.5 ± 1.2%) and *Luteolibacter* (6.8 ± 1.8%), respectively. The second most dominant bacteria groups among leeches were *Magnetospirillum* (21.4%) and Unclassified Rhodospirillaceae (15.4%), while for Gammaridae sp. they were *Rhodobacter* (18.0%) and *Leadbetterella* (18.5%). *Wolbachia* (35.4%) and Unclassified Enterobacteriaceae (13.0%) were the first and the second dominant genera for backswimmers (Fig. 8).

**Figure 9:**
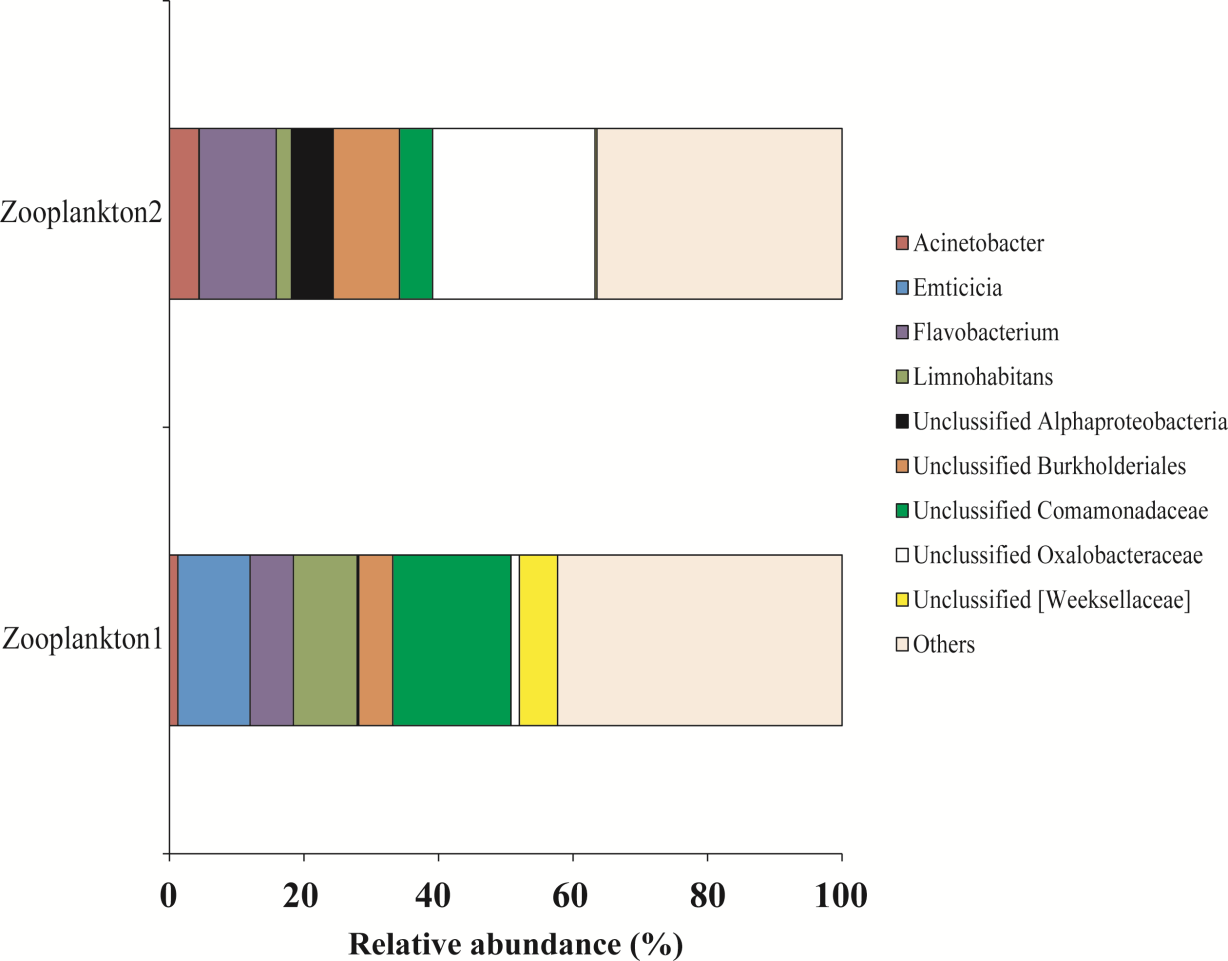
The associated microbiota of fish prey (zooplankton only) at the genus level in Teletskoye Lake.

For zooplankton crustaceans the most dominant genera were presented by Unclassified Comamonadaceae (11.3 ± 6.3%), *Flavobacterium* (8.9 ± 2.5%), and Unclassified Oxalobacteraceae (12.7 ± 11.4%) (Fig. 9).

Bacterial community of prey was significantly different from bacterial communities of all parts of gut mucosa (Table S3) and gut content (Fig. S4) of *pidschian* and *pravdinellus* (ADONIS, *p* < 0.05) but not significant when compared to the bacterial community of the environment (ADONIS, *p* ≥ 0.05).

#### Environmental microbiota

**Figure 10:**
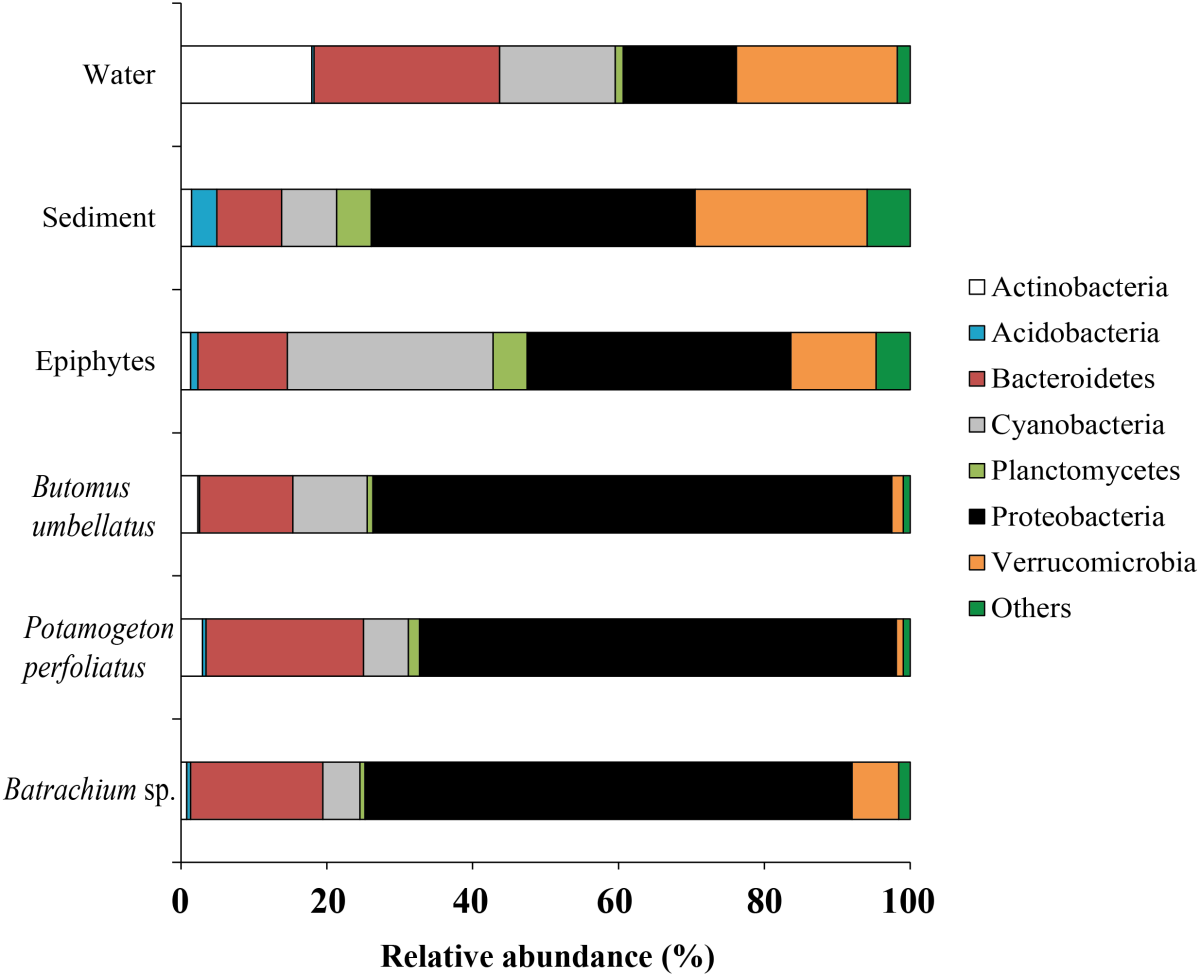
The associated microbiota of environmental compartments at phylum level in Teletskoye Lake.

Forty six bacterial phyla were identified from the associated microbiota of water, sediment, macrophytes, and stone epiphytes (Fig. 10). At the phylum level the bacterial community of macrophytes was represented by Proteobacteria and Bacteroidetes with variations in prevalence 65.4–71.2% and 12.8–21.6%, correspondingly. In the bacterial communities from stone epiphytes and sediment the phylum Proteobacteria was also a dominant taxa with prevalence of 36.2 and 44.4%, respectively; whereas the second dominant group for epiphytes was Cyanobacteria (28.2%) and for sediment –Verrucomicrobia (23.6%). In the bacterial community from water samples, five different phyla could be determined as a dominant due to their similar levels of prevalence e.g., Bacteroidetes (25.5%), Verrucomicrobia (22.1%), Actinobacteria (17.9%), and Proteobacteria (15.5%).

**Figure 11:**
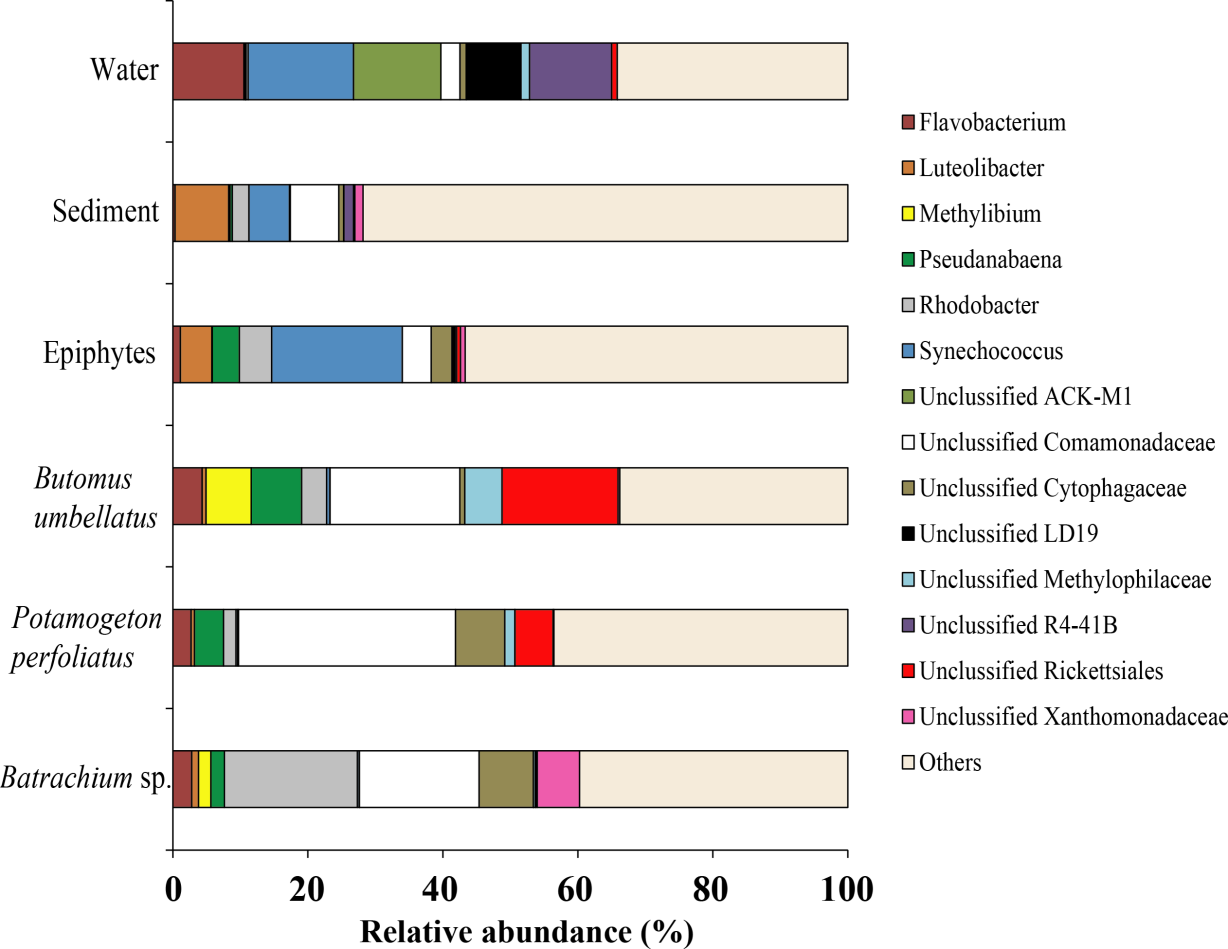
The associated microbiota of environmental compartments at the genus level in Teletskoye Lake.

Due to a high level of taxonomical variation in bacterial composition among different compartments (water, sediments, and plants) of the environment, the threshold was 5% and, thus, the largest proportion of obtained sequences was moved to the group “Others”. At the genus level, the microbiota of water was mainly composed of four taxonomical groups with quite similar levels of prevalence: *Synechococcus* (15.6%), Unclassified ACK-M1 (13.0%), Unclassified R4-41B (12.2%), and *Flavobacterium* (10.5%). The microbiota of sediment was composed of *Luteolibacter* (7.8%), Unclassified Comamonadaceae (7.2%), and *Synechococcus* (15.6%). Among macrophytes the dominant OTUs identified were Unclassified Comamonadaceae (up to 32.1%), Unclassified Rickettsiales (up to 17.2%), and *Rhodobacter* (up to 19.7%). From epiphytes, apart from “Others” there was only one dominant taxa: *Synechococcus* (19.3%) (Fig. 11).

### Comparison between gastrointestinal microbiota of fish and associated microbiota of environmental compartments

**Figure 12:**
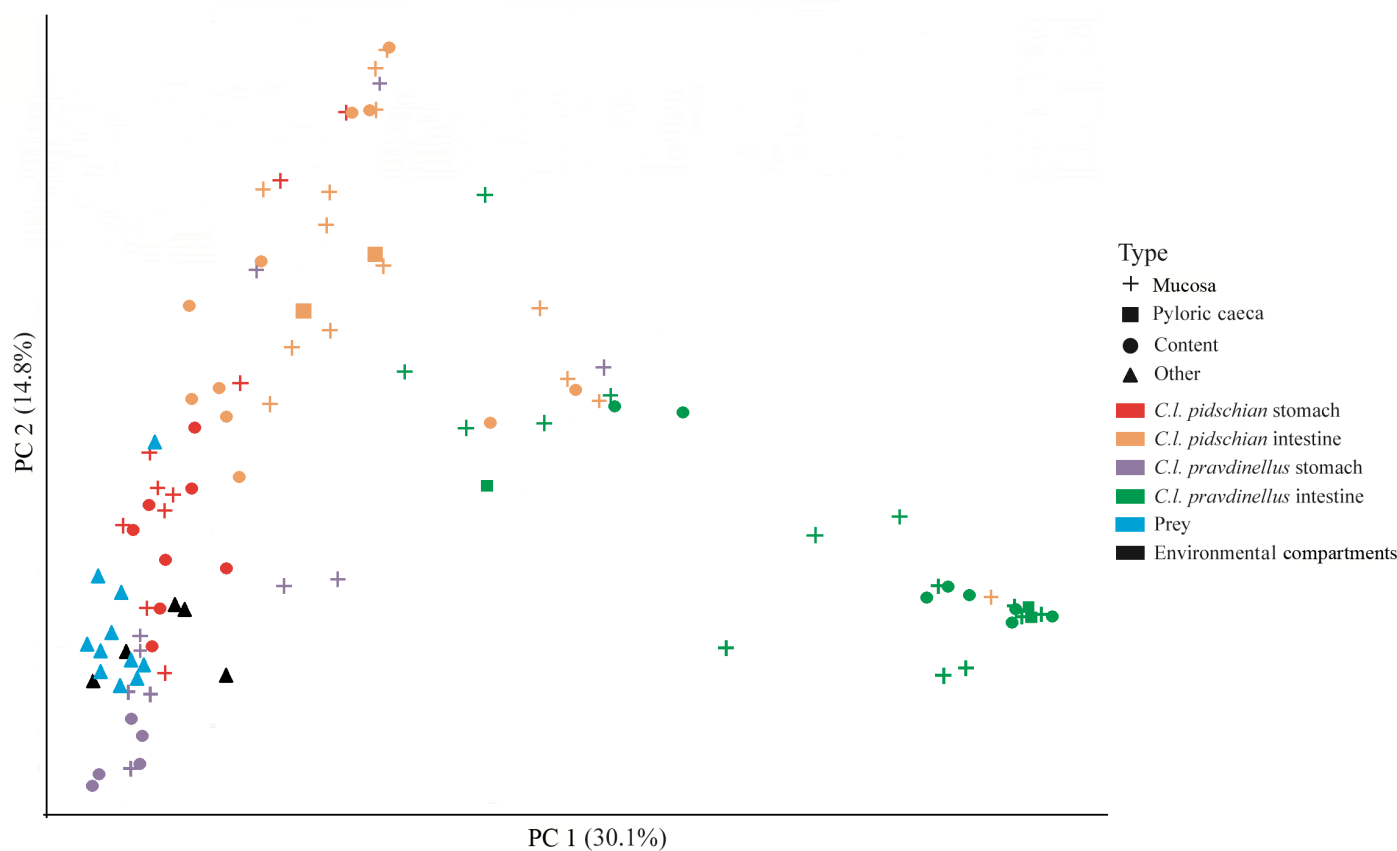
Principle coordinates analysis (PCoA) for microbiota associated with gut of fish (mucosa and content), prey and environmental compartments (water, sediments, and plants).

A scatter plot based on PCoA scores showed a grouping of the microbiota into three groups with small overlap between: (i) stomach mucosa and content of *pidschian* and *pravdinellus* as well as prey and environment (ii) intestinal mucosa and content of *C. l. pidschian*, (iii) intestinal mucosa and content of *pravdinellus* (Fig. 12). Beta diversity by prey, environment, and part of fish gut for mucosa and content is presented at Fig. S3.

#### Predicted functional metagenomes of the microbiota from fish gut using PICRUSt

PICRUSt was used to determine the microbiome functions, using OTU data to predict functional metagenomes. It showed that all groups of samples (mucosa and content from all parts of gut of *pidschian* and *pravdinellus*, environmental compartments, and prey) exhibited similar profiles of gene functions at level 1, The predicted function “metabolism” was the largest one in terms of percent among all functions, reaching to 51.7% (content from posterior intestine of *pidschian*). Such functions as “human diseases”, “organismal systems” and “none” were less than 3% overall and were not considered further. When both whitefish species were compared on level 3, the highest level of difference between *pidschian* and *pravdinellus* were found for the intestinal mucosa (75.3% of significantly different predicted functions), whereas the lowest level of difference was registered for stomach content (38.8% of significantly different predicted functions) (Table S2).

## Discussion

There are many studies focused on the gut microbiota of salmonids. However, the information about microbiota of freshwater salmonids from wild populations is very scarce as most of studies were carried out in aquaculture conditions with artificial diets (Mente et al., 2018; Michl et al., 2017). Our study provides the first detailed description of gut microbiota of a freshwater sympatric pair of Siberian *Coregonus* forms of whitefish with special emphasize on comparing the structure of the bacterial community in different parts of gut and their content separately. In our study we aimed to address the question of how the gut microbial community reflects the divergence in diet of a sympatric pair of whitefish.

### Patterns of intraspecific alpha diversity

Alpha diversity of both content and mucosa was significantly higher in stomach than in intestine in both forms of whitefish. It is well known that the stomach of gastric fish is the first part of digestive system where the food is subjected to the influence of hydrolytic enzymes and other chemical agents like hydrochloric acid. It leads to lysis of sensitive bacterial cells and subsequent bacterial DNA degradation. It is to be expected that this organ could serve as a filter that eliminates some part of the bacterial community since the bacteria associated with food are subjected to low pH levels (HCl) and the hydrolytic activity of proteases (Solovyev et al., 2018a; Ikeda-Ohtsubo et al., 2018). Li et al. (2014a) found differences along the gut in a gastric carnivorous species *Siniperca chuatsi* that may reflect the influence of a pH gradient maintained in the gut. The different abundances within the microbial community indicated that selective forces are acting within the host (Li et al., 2018). Consequently, the similarities in physiological conditions (pH, ion and gas concentrations, etc.) between both parts of the stomach and among parts of the intestine, as well as dissimilarities between stomach and intestine in the present study, is a reasonable explanation why the differences in the composition of the bacterial communities were registered for both whitefish in the same way. Indeed, we have found a significant reduction in richness and diversity estimates among samples from this study, from highest to lowest, as follows: prey > environment > stomach content > stomach mucosa > intestinal content > intestinal mucosa. We suppose that such changes in richness and diversity are explained by selective pressures of different specific conditions in the surrounding ecosystems and in the micro-environment of the fish gut. Similar results for fish gut and environmental compartments (prey, water, sediments, macrophytes) were also obtained for several fish species from Chany Lake (Kashinskaya et al., 2018). Gajardo et al. (2016) has also observed significantly lower OTU numbers as well as Chao1 and Shannon indices for bacterial communities in various parts of intestinal mucosa in comparison with content from the same digestive tract segments of *S. salar*. But Vasemägi, Visse & Kisand (2017) showed that the richness in water samples was several fold lower than in the gut microbiome of brown trout. The contradictions between our results and results obtained by Vasemägi, Visse & Kisand (2017) could be explained by differences in waterbodies where both works were done. Indeed, a lower level of diversity in the bacterial community from water in a river could be expected, as compared to in Teletsoye Lake, due to differences in flow velocity and nutrient loading in the water.

On the other hand, we have found no differences in richness and diversity of bacterial communities from mucosa and content between parts of the stomach as well as among parts of the intestine in both *pidschian* and *pravdinellus* whitefish. The lack of differences was also found for diversity estimates between mucosa and content from the same segments of the gut for both species. This suggests that differences in diet do not affect the pattern of microbial diversity along the gut of these studied whitefish. Despite this, the OTU richness of the gut between mucosa and content in every section of the gut was significantly higher for *pidschian*, which might be related to the higher OTU richness estimates observed in primary food items of *pidschian*. Thus, a higher OTU richness estimate in primary diets can lead to higher OTU richness estimates in content of the fish gut. Similar results in terms of richness estimates (Chao1 and number of OTU) among mucosa and content from different parts of the intestine were recorded for *S. salar* (Gajardo et al., 2016). Thus, we may note it as evidence of the effect of diet specialization on the OTU richness of the gut bacterial community of these studied whitefish.

### Interspecific alpha diversity

The most remarkable differences in richness and diversity estimates between *pidschian* and *pravdinellus* were found for content from different parts of the gut, whereas for mucosa the differences were less evident. Sevellec, Derome & Bernatchez (2018) found no difference in diversity estimates of intestine microbiome between the dwarf and normal *C. clupeaformis* sympatric pairs from different North American lakes. However, it has been supposed that fish with a more generalized diet (like *pidschian*) carry more diverse microbes than specialist fish species (like *pravdinellus*). Several studies have shown that the diversity of the gut microbiota of omnivorous or herbivorous fish was higher than those of carnivorous ones (Ward et al., 2009; Larsen, Mohammed & Arias, 2014; Li et al., 2018). It was found, using 29 species of cichlids within a single tribe from two lakes, that the gut microbiota in carnivores was dominated by few and abundant taxa, while in herbivores (and partly omnivores) the microbiota was distinguished by an increased bacterial richness (Baldo et al., 2017). Further, the presence of zooplankton as a food item may explain the lower values of diversity index (Shannon) in the gut of gastric paddlefish as compared to agastric bighead carp (Li et al., 2014a, Li et al., 2014b). On other hand, no differences were found for both richness and diversity among different trophic groups of fish from Chany Lake (Kashinskaya et al., 2018). In a study of stickleback and perch, diet manipulations with mixed diets demonstrated a significantly lower diversity of intestinal microbiota when compared to the microbiota of specialist fish species in two natural populations, and also in the laboratory (Bolnick et al., 2014a). At the same time, the lack of differences could be associated with features in feeding strategies of fish from different lakes. Indeed, fish in Chany Lake change their primary food items during the warmer growing season, and during some period of time the diets of different fish may be overlapping. However, the diets of *pidschian* and *pravdinellus* are stable throughout the year (Bochkarev & Zuikova, 2006). In general, these factors seem to aid the formation of a more distinct and unique mucosal microbiota in the studied whitefish forms.

### Variability of bacterial communities within the gut

In general, there is limited information on microbiota of the stomach of freshwater salmonids. This fact could be explained by low interest from researchers in this part of the fish gut due to the less important role of the stomach in relation to the microbiota, or a greater interest in focusing on nutrient uptake in the gut. In the present work we have found that the microbiota of mucosa and content of both parts of the stomach (cardiac and pyloric) were dominated by Proteobacteria, Firmicutes, Verrucomicrobia, Bacteroidetes, and Actinobacteria for both studied whitefish. The phyla Proteobacteria and Firmicutes were also registered in the stomach (mucosa and content together) of another salmonid (rainbow trout *Oncorhynchus mykiss*) reared in freshwater open cages in Ladoga Lake (Parshukov et al., 2019).

In this study, dominant bacterial taxa found in both intestinal content and mucosa, were represented at the phylum level by Proteobacteria, Firmicutes, Actinobacteria, Tenericutes and Spirochaetes for *pidschian,* and Tenericutes, Proteobacteria, Firmicutes. Actinobacteria, and Spirochaetes for *pravdinellus*. The same bacterial phyla (except Spirochaetes) were identified in intestinal mucosa for different sympatric pairs of congeneric species *C. clupeaformis* from different lakes (Sevellec, Derome & Bernatchez, 2018). Proteobacteria and Firmicutes were also mentioned as core intestinal microbiota (in mucosa, content or combined samples) of other freshwater salmonids such as rainbow trout, *O. mykiss* (Wong et al., 2013; Lyons et al., 2016; Parshukov et al., 2019), brown trout *S. trutta* (Vasemägi, Visse & Kisand, 2017), and Atlantic salmon, *S. salar* (He, Chaganti & Heath, 2018). Moreover, these phyla were identified as core microbiota of non-salmonid freshwater fishes (Sullam et al., 2012; Li et al., 2014a; Ye et al., 2014; Baldo et al., 2015; Kashinskaya et al., 2015; Liu et al., 2016; Kashinskaya et al., 2018). Such similarities in dominant phyla are likely to suggest that both these phyla are typical for many fishes regardless of their taxonomical position, feeding habits, ambient water conditions, etc.

The lack of differences observed for mucosa and content of both studied whitefish forms, between different parts of the stomach as well as among different sections of the intestine could be explained by similarities in biochemical composition of the media within the lumen of these parts of gut. Our study showed a significant effect for the type of sample (mucosa or content) on the gut bacterial composition for *pidschian* and no effect for *pravdinellus*. On other hand, the values of R^2^ (size effect) for both whitefish were very low meaning that the influence of this factor is actually absent or negligibly low (Anderson, 2001). In our previous work on the Prussian carp *Carassius gibelio*, Crucian carp *C. carassius*, Common carp *Cyprinus carpio*, roach *Rutilus rutilus*, dace *Leuciscus leuciscus*, ide *L. idus*, perch *Perca fluviatilis*, and pike-perch *Sander lucioperca* there was found a significant effect from sample type (Kashinskaya et al., 2017). Such differences among gastric fish could be explained by features of the studied waterbodies. Chany Lake is characterized as a shallow lake (average depth is 2 m) without a thermocline whereas in Teletskoye Lake the average depth is 187 m with a strong thermocline. Therefore, in conditions of Teletskoye Lake the fish may choose the preferred water condition, such as temperature, concentration of dissolved oxygen and so on, whereas in Chany Lake displacement along a horizontal plane (due to shallow depth) offers very limited variation in water quality. Moreover, Vasemägi, Visse & Kisand (2017) has also found significant associations of the gut bacterial composition of brown trout with water temperature, dissolved oxygen, and geomorphology in different rivers. On the other hand, we may hypothesize that the stomach, as a physicochemical barrier (acid digestion), reduces the diversity of the bacterial community, as the community transitions from the stomach to the intestine and becomes more similar to the microbiota of the intestinal mucosa.

It was previously shown that the composition of the bacterial community at the lowest taxonomic level is very diverse even within the same species (Sullam et al., 2012; Vasemägi, Visse & Kisand, 2017; Parshukov et al., 2019). Indeed, the genera occurring at high levels of abundance in the intestine of different salmonids have been identified as the following: brown trout—*Yersinia*, *Shigella*, and *Serratia* (Enterobacteria) (Vasemägi, Visse & Kisand, 2017); for rainbow trout from different studies—*Mycoplasma*, *Brevinema*, *Lactobacillus*, *Acetanaerobacterium*, *Catellicoccus*, *Streptococcus*, *Weissella*, *Leuconostoc*, *Lactococcus*, *Enterococcus*, and *Bacillus* (Lyons et al., 2016) *Lactobacillus*, *Streptococcus*, *Staphylococcus*, *Clostridia* (Wong et al., 2013) *Streptophyta*, *Bacillus*, *Serratia*, *Cetobacterium*, *Pseudomonas* and bacteria from the order Rickettsiales (Parshukov et al., 2019); for Atlantic salmon—*Pseudomonas*, *Acinetobacter*, *Deefgea*, *Rhodobacter*, *Flavobacterium*, and *Lactobacillus* (He, Chaganti & Heath, 2018). In general, such high variability in results obtained by different research groups may be explained by the influence of water and holding conditions (Vasemägi, Visse & Kisand, 2017; Dehler, Secombes & Martin, 2017), diets (Ringo et al., 2016), DNA extraction protocols (Kashinskaya et al., 2017), high level of intraspecific variation (Bolnick et al., 2014a; Vasemägi, Visse & Kisand, 2017), sex (Bolnick et al., 2014a), fish length and mass (Vasemägi, Visse & Kisand, 2017), time after feeding (Zhang et al., 2017) and many others factors that are typically speculated in studies focused on gut bacterial communities. Therefore, we did not try to explain the variability of gut microbiota among fish from different geographical regions and reared in diverse conditions due to the previously mentioned restrictions. Thus, with the knowledge currently available, there is no value in comparison of the taxonomical composition of the gut microbiota of fish found by different research groups for the aims of the present study.

### Interspecific differences in bacterial communities

In the present work we have found remarkable differences in composition of the bacterial community between *pidschian* and *pravdinellus*, for their gut mucosa as well as gut content. In several previous studies it has been shown that different species of fish inhabiting the same waterbody may possess significantly different gut microbial communities (Li et al., 2012; Li et al., 2014a). Many studies have focused on factors that may determine the formation of a gut bacterial community (Roeselers et al., 2011; Sullam et al., 2012; Dehler, Secombes & Martin, 2017). It is widely accepted that the diet is likely to be the main driver forming it (see review Ringo et al., 2016) and a similar microbiota structure may represent a similar dietary habit (Li et al., 2018). From our point of view the important role in the shaping of the bacterial community belongs to the features of the digestive physiology of fish. We did not find differences in distribution of key digestive enzymes (stomach, pancreatic and brush border) along the length of the gut or in zymograms of alkaline proteases, but the specific activity of all pancreatic enzymes was significantly higher in the intestine of *pidschian* than in *pravdinellus* (Solovyev et al., 2018b). It means that food items belonging to different taxonomical groups (mollusks, gammarids, cladocera, copepod, larva of trichoptera, etc.) and consequently their body as characterized by different proximate composition (e.g., lipids, proteins, sugars) will be hydrolyzed by the same digestive enzymes acting on different biochemical components such as poly and oligopeptides, sugars, metal ions (for example Ca^2+^ from mollusks shells), etc. As these components are digested they convert the contents of the luminal space of the intestine into a specific selective media for growing specific groups of bacteria. It is widely accepted that different biochemical components promote the growth of different bacteria. This assumption may explain the total absence of the phylum Tenericutes in the stomach content of *pravdinellus,* but its appearance as a dominant in the intestinal content. If it is taken into account that Tenericutes was also a dominant phylum in the intestinal mucosa, we may suppose that this group of bacteria, once established in the mucosa, can then serve as a source for colonization to the gut content.

### Gut microbiota and environmental bacterial communities

In this study, we have found that the microbiota of stomach mucosa, stomach content, prey and environmental compartments formed one cluster on scatter plot (Fig. 12), but according to the ADONIS analysis these similarities were significant only between prey/compartments and mucosa/content of cardiac stomach for *pidschian*. These similarities may be explained by that fact that this part of the gut is the first to be contacted continuously (after oesophagus) with environmental constituents and prey. Hence, the microbiota of prey and environment in the cardiac stomach are not so changed under the specific conditions of the internal medium of other parts of the gut and, thus, have higher levels of similarity with the original bacterial load that had entered. Sevellec, Derome & Bernatchez (2018) also did not find considerable association between water microbiota and fish intestinal microbiota, while the bacterial communities from five examined lakes were very different. However, in his work the stomach microbiota was not analyzed. In many studies there has not been found a correlation between bacterial taxonomic composition of fish gut and that of environmental compartments from the wild (water, sediment, food items, etc.) (Li et al., 2014a; Schmidt et al., 2015; Llewellyn et al., 2016; Vasemägi, Visse & Kisand, 2017; He, Chaganti & Heath, 2018; Kashinskaya et al., 2015; Kashinskaya et al., 2018). Further, it was shown that food-associated microbes drive gut microbial community diversity to a greater extent than water-associated microbes (Bolnick et al., 2014a; Smith et al., 2015). On the other hand, it is without doubt that the microbiota of the fish gut were harvested from environmental compartments, but the incoming microbial community is transformed in unpredictable ways under the specific conditions of different parts of the fish gut (He, Chaganti & Heath, 2018; Kashinskaya et al., 2015; Kashinskaya et al., 2018). Indeed, the present study supports this model since the stomach mucosa and content with prey and environment are located closer to each other on the scatter plot based on PCoA scores, whereas the microbial communities of intestine are much more distinct.

### Predicted functions of microbiome

Differences in bacterial communities may lead to differences in the functional role of the communities. During recent years, the software PICRUSt has appeared as an approach applied to estimate the predicted functions of given bacterial communities (Langille et al., 2013; Lyons et al., 2016; Kashinskaya et al., 2018). Using the approach of PICRUSt, we have checked the hypothesis that taxonomically different bacterial communities from the gut of *pidschian* and *pravdinellus* will demonstrate parallelism in their functionality. We have found great differences in predicted functions between bacterial communities from intestinal content and mucosa of *pidschian* and *pravdinellus,* especially regarding to genes connected to metabolism. It has sense that a bacterial community with a specialized taxonomic composition should also be specialized in functionality. Thus, our hypothesis is not supported by obtained results and the bacterial communities of the studied whitefish demonstrate differences in their functional role within their respective hosts.

Many bacterial species and strains produce enzymes used for fish digestive functions, thus, playing a significant role in digestion and metabolism of a broad range of nutrients (see review Ray, Ghosh & Ringø, 2012). As was found for cellulase (Li et al., 2018), in our study the same role may be attributed to chitin content since studied whitefish forms feed on many invertebrates whose composition includes a significant proportion of chitin. It may be expected to find higher level of chitin in the diet of *pravdinellus* since this fish feeds only on crustaceans. Indeed, chitinase activity was determined in stomach and pyloric caeca of different fish (see review Ray, Ghosh & Ringø, 2012) including salmonids (Askarian et al., 2012), but it is known that salmonids seem to have a low ability to utilize chitin themselves. On other hand, from fish gut there were identified many different bacteria that may hydrolyze chitin (Ringo et al., 2012). In our work such genera as *Acinetobacter* (from mucosa), *Flavobacterium* (from mucosa and content), and *Bacillus* (from intestinal content) could be designated as potential producers of chitinases (Ringo et al., 2012) and they were dominant in the stomach of *pravdinellus,* whereas the prevalence of these genera in the stomach of *pidschian* was lower. At the same time, the prevalence of *Acinetobacter* was relatively high in pyloric caeca of *pidschian*. The cellulolytic bacterial community, including *Aeromonas*, *Enterobacter*, *Enterococcus*, *Citrobacter*, *Bacillus*, *Raoultella*, *Vibrio* and some unclassified bacteria, were dominant in the guts of herbivorous fish (Ray, Ghosh & Ringø, 2012; Liu et al., 2016). In this study there were also identified several microbial taxa that contributed to enzyme production in the gut (such as *Aeromonas*, *Clostridium*, *Enterobacter*, and *Vibrio*) in all samples (Ray, Ghosh & Ringø, 2012). With such specialized bacteria present in the gut of each host that have seemingly particular niches producing diet-specific enzymes, the influence to the microbiota of each form of whitefish should also be evident. Further studies are needed to investigate the exogenous and endogenous factors that influence the enzymatic activity of hybrids and their parents. Moreover, we believe that different specific digestive enzymes synthesized by species-specific bacterial communities hydrolyze a broad range of substrates. The metabolic by-products of gut bacteria can have profound influence on the host physiology (Mazzoli, Riedel & Pessione, 2017; Martin et al., 2019). Specific molecules, for example short peptides, released during such enzymatic activity could function as “inter-kingdom” signaling molecules for host cells (Strandwitz et al., 2019). Thus, a species-specific bacterial community might possess specific digestive enzymes that drive adaptation processes of its host or even lead to symbiotic relations between some taxa and their host (Lyons et al., 2017).

### Parasite infection

As mentioned above, the gut bacterial community is affected by a large number of abiotic and biotic factors. One such factor could be parasitic infections since it was shown that gut parasites (mainly cestodes) could affect fish digestion (Izvekova & Solovyev, 2016). However, researchers do not always consider the presence of parasites in studies of the microbiota of fish gut, whereas fish from natural waterbodies may be infected by different species of helminths e.g., cestodes, trematodes, acanthocephalans with high levels of prevalence. Indeed, it was shown that parasites are an important part of biological energy flow in natural ecosystems and researchers have stated the important role of such parasites (Kanaya et al., 2019). At the same time, if studies are focused on ecosystem factors (other than parasites) that influence the biology or physiology of fishes, the parasites “disappear” from study design as a possible factor and the potential infection-induced changes in the host organism are not examined within the same context. First, we think that with a low-grade infestation, which occurs more frequently in the wild, helminthes rarely induce obvious pathogenic effects in their hosts and such fish are marked as “healthy”. Therefore, researchers do not take into account the possible influence of helminthes on the studied bacterial community. Second, due to a small body size of some helminth species, they are often overlooked by non-parasitologists. Third, the researches usually tend to choose the fish harboring no helminths for their studies, considering them to be closer to a physiological norm, but such approach drives the study far from reality since the host-parasite phenome is a coevolved system with very putative intimate relationships, whereas a parasite-free host could be more physiologically abnormal than an infected host. Along with this last point is that some researchers just ignore the presence of parasites in the fish gut. For salmonid fishes there have been described many taxonomically different groups of parasites infecting their gut sometimes in great amount. For whitefish from Teletskoye Lake there have been described several parasites infecting the gut (Bochkarev & Gafina, 1993). In the present study, we have found cestodes in the intestines of *pravdinellus*. From our present data we cannot state what the possible influence on the gut bacterial community might be due to the low number of infected and uninfected fish examined; therefore a more focused study on this topic in the future is needed.

## Conclusion

The differences in feeding habits as a main force in evolutionary divergence of whitefish in oligotrophic lakes like Teletskoye Lake, have demonstrated the deep effect on the composition and predicted functionality of the gut bacterial community. On the one hand, the gut microbiota of this sympatric pair of whitefish demonstrated similarities in richness and diversity estimates. It means that the factors that shape the bacterial communities along the gut of both whitefish act in the same way regardless of the differences in their feeding habits. In part, this likely correlates with similarities in physiological conditions (pH, ion and gas concentrations, etc.) along the length of the gut of whitefish. On other hand, the dissimilarities in taxonomical composition from the gut were remarkable. This suggests that the diet shapes the taxonomical composition as well as the functionality of gut bacterial community in completely different ways. Moreover, our findings have revealed the potential importance of microbial communities from the fish stomach to interpret the relationships among bacterial communities from fish gut and environmental compartments that has been largely ignored in previous studies. Hence, the comparative analysis of microbial communities among the stomach and other parts of the gut provides additional views to understand the mechanisms underlying the shaping of the intestinal microbiota.

##  Supplemental Information

10.7717/peerj.8005/supp-1 Figure S1 The rarefaction curves for all samples

10.7717/peerj.8005/supp-2 Figure S2 Beta diversity for each part of the gut (mucosa and content inclusive)

10.7717/peerj.8005/supp-3 Figure S3 Beta diversity by prey, environmental compartments, and part of fish gut for mucosa and content

10.7717/peerj.8005/supp-4 Figure S4 Venn diagrams of OTU membership * - Percentage of unique OTUs calculated from the total number of OTUs

10.7717/peerj.8005/supp-5 Table S1 Results of pairwise comparisons for all studied groups of samples in alpha-diversity measures (Mann–Whitney pairwise test) *Compartments –environmental compartments

10.7717/peerj.8005/supp-6 Table S2 The presence (+) or absence (−) of significant differences at *P* ≤ 0.05 (ANOSIM) in predicted functions of gut bacterial community at level 1, 2, and 3 between *C. lavaretus pidschian* and *C. lavaretus pravdinellus*

10.7717/peerj.8005/supp-7 Table S3 Results of pairwise comparisons of *C. l. pidschian* and *C.l. pravdinellus* mucosa from different parts of gut, environmental compartments, and prey ¥*C. l. pidshian*/*C.l. pravdinellus*; *compartments –environmental compartments

10.7717/peerj.8005/supp-8 Table S4 Results of pairwise comparisons of *C. l. pidschian* and *C.l. pravdinellus* content from different parts of gut, environmental compartments, and prey ¥*C. l. pidschian* /*C. l. pravdinellus*; *compartments –environmental compartments

10.7717/peerj.8005/supp-9 Table S5A The effect (ADONIS) of parts of gut and sample type (mucosa or content) on *C. l. pidschian* and *C. l. pravdinellus* microbiota ¥*C. l. pidschian* /*C. l. pravdinellus*

10.7717/peerj.8005/supp-10 Table S5B The pairwise comparisons of different parts of gut (mucosa and content together) for *C. l. pidschian* and *C. l. pravdinellus*¥*C. l. pidschian* /*C. l. pravdinellus*

10.7717/peerj.8005/supp-11 Table S6A Results of the effect (ADONIS) of forms and part of gut for mucosa and content on *C. l. pidschian* and *C. l. pravdinellus* microbiota ¥Mucosa/Content

10.7717/peerj.8005/supp-12 Table S6B Pairwise comparisons for mucosa from different parts of gut of *C. l. pidschian* and *C.l. pravdinellus* textyen *C.l.*–*C. l. pidschian*;* C.l.p.*–*C. l. pravdinellus*

10.7717/peerj.8005/supp-13 Table S7 Pairwise comparisons for content from different parts of gut of *C. l. pidschian* and *C. l. pravdinellus* textyen *C.l.*–*C. l. pidschian*;* C.l.p.*–*C. l. pravdinellus*

## Additional Information and Declarations

### Data Availability

The following information was supplied regarding data availability: The raw data associated with bacterial diversity and PICRUSt analysis of all studied samples are available at the Sequence Read Archive: PRJNA526436 and PRJNA526346.
