# Supplementary figures and images for: The effect of diet on the structure of gut bacterial community of sympatric pair of whitefishes (Coregonus lavaretus): one story more
Source: PeerJ. 2019 Dec 3;7:e8005. doi: 10.7717/peerj.8005 (PMC6896945; doi:10.7717/peerj.8005)

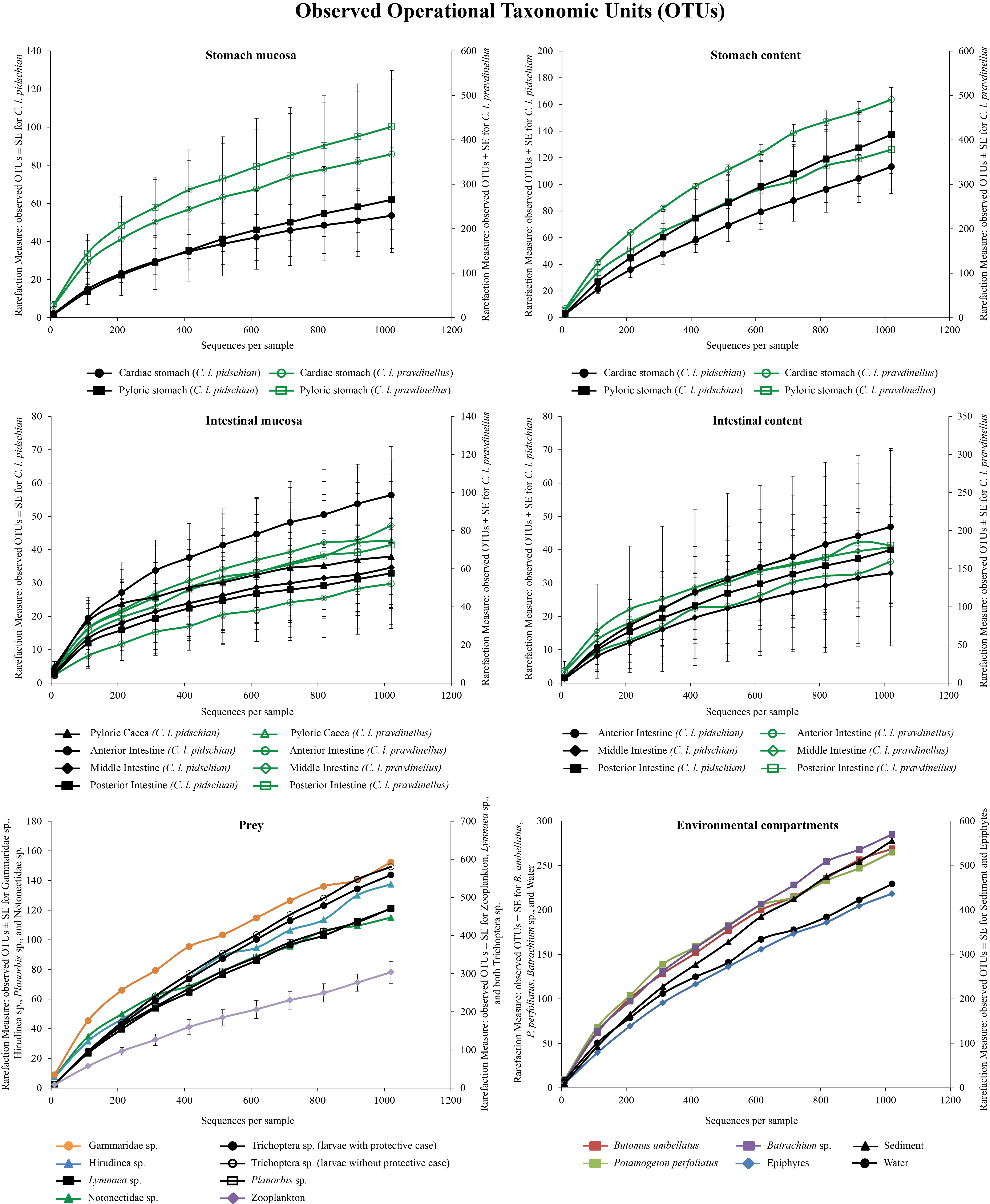

Supplement: Figure S1 [file peerj-07-8005-s001.png]

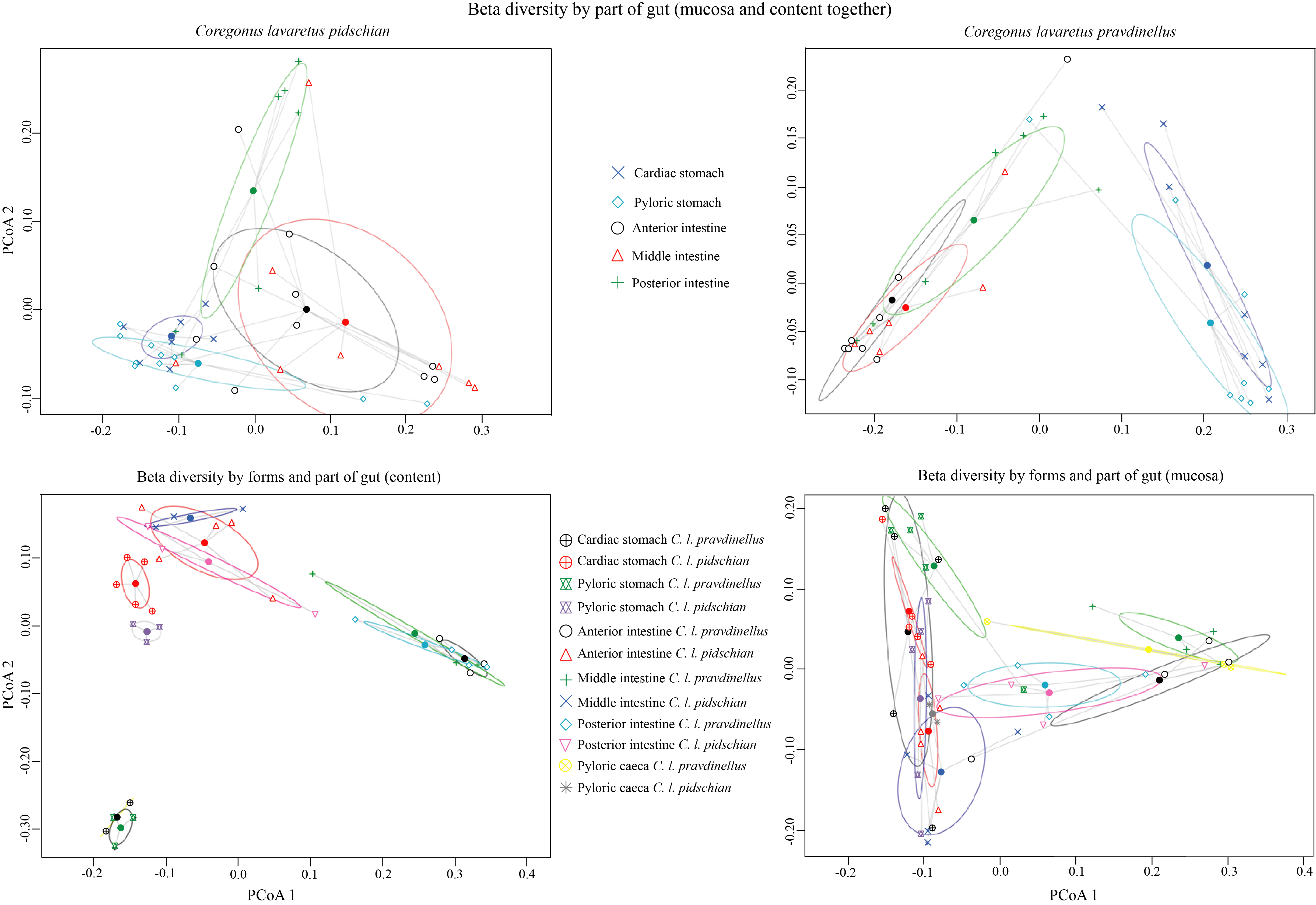

Supplement: Figure S2 [file peerj-07-8005-s002.png]

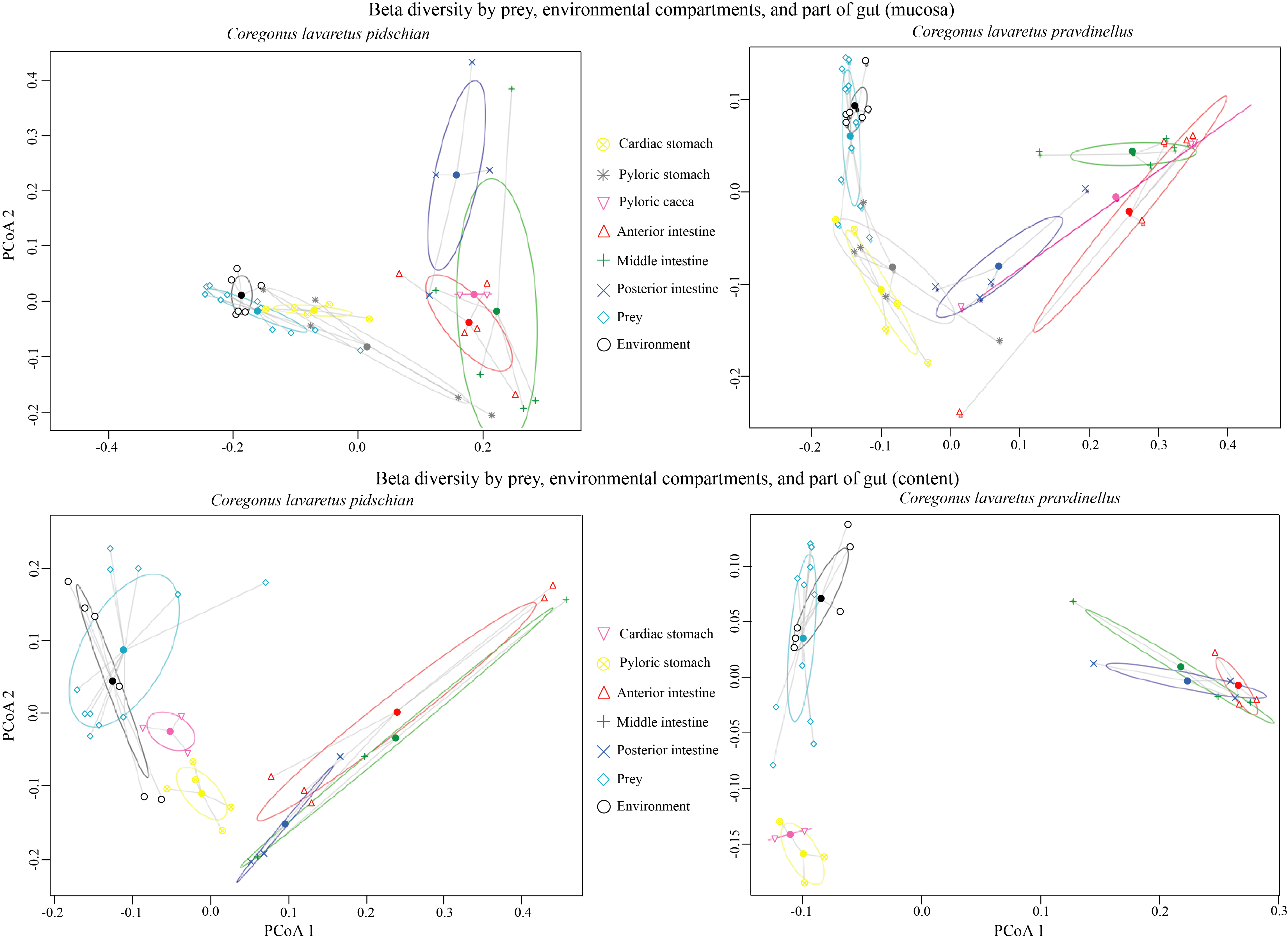

Supplement: Figure S3 [file peerj-07-8005-s003.png]

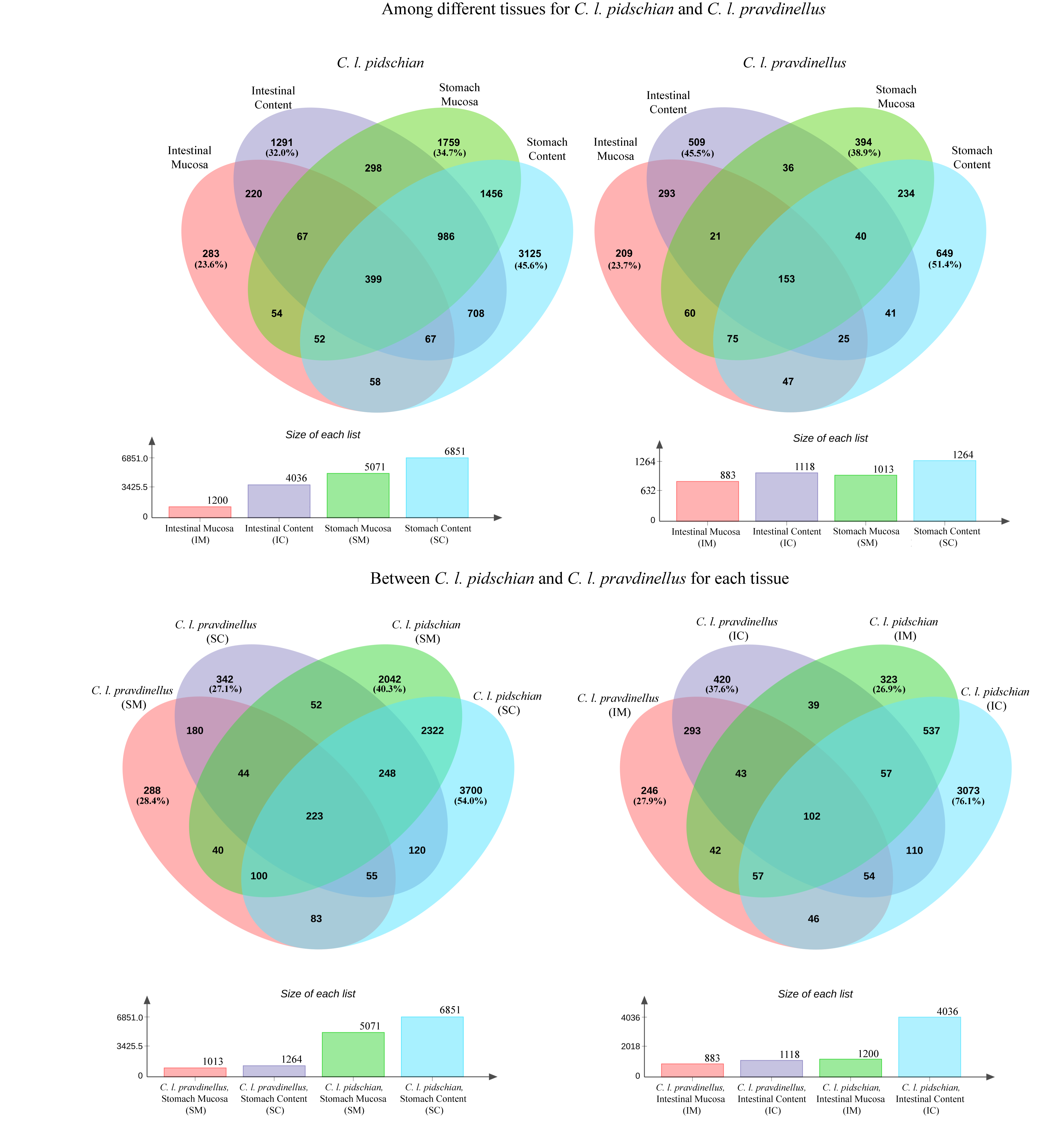

Supplement: Figure S4 — * - Percentage of unique OTUs calculated from the total number of OTUs [file peerj-07-8005-s004.png]

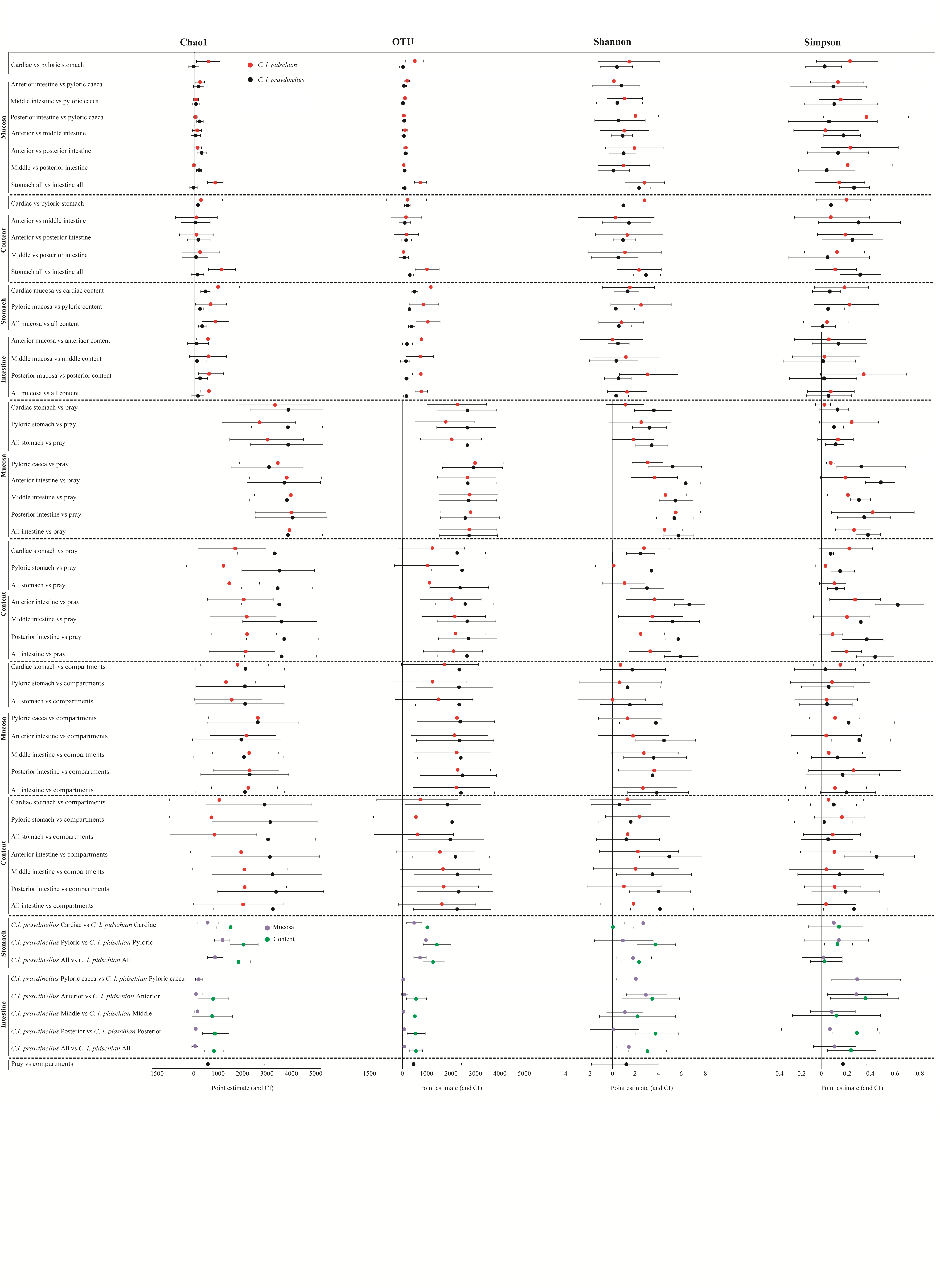

Supplement: Table S1 — *Compartments –environmental compartments [file peerj-07-8005-s005.png]
