# Supplementary material for: The effect of diet on the structure of gut bacterial community of sympatric pair of whitefishes (Coregonus lavaretus): one story more
Source: PeerJ. 2019 Dec 3;7:e8005. doi: 10.7717/peerj.8005 (PMC6896945; doi:10.7717/peerj.8005)
Supplement: Table S3 — ¥C. l. pidshian/C.l. pravdinellus; *compartments –environmental compartments [file peerj-07-8005-s007.docx]

| **Comparison** | **ADONIS** | | **Homogeneity of multivariate dispersions** |
| --- | --- | --- | --- |
|  | **R^2^** | **FDR P-value** | **Permuted p-value** |
| Compartments* vs Anterior intestine | 0.41/0.54^¥^ | **0.014**/**0.009** | 0.931/0.820 |
| Compartments vs Middle intestine | 0.37/0.61 | **0.014**/**0.013** | 0.082/0.108 |
| Compartments vs Posterior intestine | 0.32/0.35 | **0.020**/**0.015** | **0.002**/**0.011** |
| Compartments vs Prey | 0.07/0.07 | 0.389/0.366 | 0.748/0.714 |
| Compartments vs Pyloric caeca | 0.44/0.51 | 0.065/**0.023** | 0.050/0.364 |
| Compartments vs Cardiac stomach | 0.19/0.27 | 0.078/**0.009** | 0.662/0.161 |
| Compartments vs Pyloric stomach | 0.23/0.30 | **0.020**/**0.009** | 0.257/0.607 |
| Anterior intestine vs Middle intestine | 0.11/0.08 | 0.416/0.865 | 0.155/0.409 |
| Anterior intestine vs Posterior intestine | 0.21/0.28 | 0.078/0.051 | **0.003**/0.357 |
| Anterior intestine vs Prey | 0.35/0.52 | **0.005**/**0.003** | 0.716/0.626 |
| Anterior intestine vs Pyloric caeca | 0.14/0.04 | 0.762/1.000 | 0.109/0.791 |
| Anterior intestine vs Cardiac stomach | 0.28/0.45 | **0.021**/**0.015** | 0.708/0.626 |
| Anterior intestine vs Pyloric stomach | 0.19/0.46 | 0.164/**0.027** | 0.388/0.936 |
| Middle intestine vs Posterior intestine | 0.16/0.35 | 0.306/0.074 | 0.149/**0.008** |
| Middle intestine vs Prey | 0.33/0.57 | **0.005**/**0.004** | **0.034**/0.139 |
| Middle intestine vs Pyloric caeca | 0.19/0.08 | 0.389/0.831 | 0.073/0.144 |
| Middle intestine vs Cardiac stomach | 0.28/0.55 | **0.021**/**0.018** | 0.095/**0.040** |
| Middle intestine vs Pyloric stomach | 0.18/0.56 | 0.133/**0.015** | 0.470/0.150 |
| Posterior intestine vs Prey | 0.30/0.33 | **0.005**/**0.004** | **0.002**/**0.006** |
| Posterior intestine vs Pyloric caeca | 0.21/0.25 | 0.416/0.243 | **0.005**/0.366 |
| Posterior intestine vs Cardiac stomach | 0.27/0.26 | **0.021**/**0.027** | **0.001**/0.264 |
| Posterior intestine vs Pyloric stomach | 0.24/0.28 | **0.021**/**0.048** | **0.023**/0.167 |
| Prey vs Pyloric caeca | 0.33/0.45 | **0.024**/**0.009** | 0.106/0.214 |
| Prey vs Cardiac stomach | 0.12/0.20 | 0.079/**0.009** | 0.908/0.077 |
| Prey vs Pyloric stomach | 0.18/0.23 | **0.020**/**0.004** | 0.162/0.397 |
| Pyloric caeca vs Cardiac stomach | 0.38/0.41 | 0.078/**0.030** | 0.208/0.880 |
| Pyloric caeca vs Pyloric stomach | 0.31/0.42 | 0.133/**0.048** | 0.069/0.751 |
| Cardiac stomach vs Pyloric stomach | 0.12/0.12 | 0.416/0.426 | 0.278/0.573 |
