# Supplementary material for: The effect of diet on the structure of gut bacterial community of sympatric pair of whitefishes (Coregonus lavaretus): one story more
Source: PeerJ. 2019 Dec 3;7:e8005. doi: 10.7717/peerj.8005 (PMC6896945; doi:10.7717/peerj.8005)
Supplement: Table S4 — ¥C. l. pidschian /C. l. pravdinellus; *compartments –environmental compartments [file peerj-07-8005-s008.docx]

| **Comparison** | **ADONIS** | | **Homogeneity of multivariate dispersions** |
| --- | --- | --- | --- |
|  | **R^2^** | **FDR P-value** | **Permuted p-value** |
| *Compartments vs Anterior intestine | 0.33/0.64^¥^ | **0.014**/**0.028** | **0.045**/**0.001** |
| Compartments vs Middle intestine | 0.32/0.49 | **0.028**/**0.028** | 0.210/0.848 |
| Compartments vs Posterior intestine | 0.27/0.50 | 0.053/**0.028** | 0.832/0.923 |
| Compartments vs Prey | 0.07/0.07 | 0.338/0.387 | 0.885/0.982 |
| Compartments vs Cardiac stomach | 0.21/0.34 | 0.109/0.072 | 0.081/**0.002** |
| Compartments vs Pyloric stomach | 0.26/0.43 | **0.016**/**0.028** | **0.014**/**0.005** |
| Anterior intestine vs Middle intestine | 0.09/0.19 | 0.749/0.525 | 0.931/**0.016** |
| Anterior intestine vs Posterior intestine | 0.18/0.21 | 0.338/0.442 | 0.311/**0.041** |
| Anterior intestine vs Prey | 0.28/0.54 | **0.006**/**0.017** | 0.150/**0.002** |
| Anterior intestine vs Cardiac stomach | 0.28/0.93 | 0.131/0.131 | **0.020**/0.238 |
| Anterior intestine vs Pyloric stomach | 0.26/0.91 | **0.023**/0.131 | **0.002**/0.103 |
| Middle intestine vs Posterior intestine | 0.17/0.08 | 0.800/0.900 | 0.575/0.944 |
| Middle intestine vs Prey | 0.26/0.43 | **0.014**/**0.017** | 0.353/0.859 |
| Middle intestine vs Cardiac stomach | 0.34/0.69 | 0.131/0.131 | 0.121/0.093 |
| Middle intestine vs Pyloric stomach | 0.29/0.71 | **0.042**/0.131 | **0.024**/0.056 |
| Posterior intestine vs Prey | 0.23/0.44 | **0.014**/**0.017** | 0.907/0.924 |
| Posterior intestine vs Cardiac stomach | 0.30/0.67 | 0.131/0.131 | 0.309/0.126 |
| Posterior intestine vs Pyloric stomach | 0.23/0.70 | 0.099/0.131 | 0.126/0.092 |
| Prey vs Cardiac stomach | 0.12/0.19 | 0.109/**0.028** | 0.230/**0.013** |
| Prey vs Pyloric stomach | 0.19/0.28 | **0.014**/**0.017** | 0.091/**0.016** |
| Cardiac stomach vs Pyloric stomach | 0.20/0.25 | 0.285/0.442 | 0.702/0.473 |
