# Supplementary material for: The effect of diet on the structure of gut bacterial community of sympatric pair of whitefishes (Coregonus lavaretus): one story more
Source: PeerJ. 2019 Dec 3;7:e8005. doi: 10.7717/peerj.8005 (PMC6896945; doi:10.7717/peerj.8005)
Supplement: Table S5A — ¥C. l. pidschian /C. l. pravdinellus [file peerj-07-8005-s009.docx]

| **Factor** | **Df** | **Sums of Sqs** | **Mean Sqs** | **F. Model** | **R^2^** | **Pr (>F)** |
| --- | --- | --- | --- | --- | --- | --- |
| Part of gut | 4/4^¥^ | 0.70/1.28 | 0.17/0.32 | 3.03/8.12 | 0.24/0.50 | **0.0001/0.0001** |
| Sample type | 1/1 | 0.12/ 0.07 | 0.12/0.07 | 2.07/1.84 | 0.04/0.03 | **0.04/**0.12 |
| Part of gut* Sample type | 4/4 | 0.16/ 0.15 | 0.04/0.04 | 0.69/0.92 | 0.06/0.06 | 0.94/0.51 |
