# Supplementary material for: The effect of diet on the structure of gut bacterial community of sympatric pair of whitefishes (Coregonus lavaretus): one story more
Source: PeerJ. 2019 Dec 3;7:e8005. doi: 10.7717/peerj.8005 (PMC6896945; doi:10.7717/peerj.8005)
Supplement: Table S5B [file peerj-07-8005-s010.docx]

| **Comparison** | **ADONIS** | | **Homogeneity of multivariate dispersions** |
| --- | --- | --- | --- |
|  | **R^2^** | **FDR P-value** | **Permuted p-value** |
| Anterior intestine vs Middle intestine | 0.06/0.06^¥^ | 0.358/0.509 | 0.115/ 0.766 |
| Anterior intestine vs Posterior intestine | 0.15/0.18 | **0.016**/**0.039** | **0.019**/ **0.021** |
| Anterior intestine vs Cardiac stomach | 0.23/0.52 | **0.001**/**0.001** | 0.373/ 0.063 |
| Anterior intestine vs Pyloric stomach | 0.16/0.57 | **0.019**/**0.001** | 0.742/ 0.338 |
| Middle intestine vs Posterior intestine | 0.14/0.14 | 0.053/0.106 | 0.497/ **0.004** |
| Middle intestine vs Cardiac stomach | 0.25/0.51 | **0.001**/**0.001** | **0.041**/ **0.041** |
| Middle intestine vs Pyloric stomach | 0.19/0.56 | **0.011**/**0.002** | 0.157/ 0.390 |
| Posterior intestine vs Cardiac stomach | 0.21/0.33 | **0.001**/**0.001** | **0.005**/ 0.388 |
| Posterior intestine vs Pyloric stomach | 0.19/0.38 | **0.004**/**0.001** | 0.057/ 0.069 |
| Cardiac stomach vs Pyloric stomach | 0.07/0.10 | 0.261/0.255 | 0.764/ 0.309 |
