# Supplementary material for: The effect of diet on the structure of gut bacterial community of sympatric pair of whitefishes (Coregonus lavaretus): one story more
Source: PeerJ. 2019 Dec 3;7:e8005. doi: 10.7717/peerj.8005 (PMC6896945; doi:10.7717/peerj.8005)
Supplement: Table S6A — ¥Mucosa/Content [file peerj-07-8005-s011.docx]

| **Factor** | **Df** | **Sums of Sqs** | **Mean Sqs** | **F. Model** | **R^2^** | **Pr (>F)** |
| --- | --- | --- | --- | --- | --- | --- |
| Forms | 1/1^¥^ | 0.41/0.42 | 0.41/0.42 | 8.59/13.84 | 0.11/0.22 | **0.0001/0.0001** |
| Part of gut | 5/4 | 0.70/0.49 | 0.14/0.12 | 2.93/4.06 | 0.19/0.25 | **0.0001/0.0001** |
| Forms*Part of gut | 5/4 | 0.58/0.34 | 0.12/0.08 | 2.41/2.80 | 0.16/0.17 | **0.0002**/**0.0004** |
