# Supplementary material for: The effect of diet on the structure of gut bacterial community of sympatric pair of whitefishes (Coregonus lavaretus): one story more
Source: PeerJ. 2019 Dec 3;7:e8005. doi: 10.7717/peerj.8005 (PMC6896945; doi:10.7717/peerj.8005)
Supplement: Table S6B — textyen C.l.–C. l. pidschian; C.l.p.–C. l. pravdinellus [file peerj-07-8005-s012.docx]

| **Comparison** | **ADONIS** | | **Homogeneity of multivariate dispersions** |
| --- | --- | --- | --- |
|  | **R^2^** | **FDR P-value** | **Permuted P-value** |
| ^¥^Anterior intestine**C.l.p.* vs Anterior intestine* *C.l.* | 0.47 | **0.043** | 0.969 |
| Anterior intestine**C.l.p.* vs Middle intestine**C.l.p.* | 0.09 | 0.805 | 0.417 |
| Anterior intestine**C.l.p.* vs Middle intestine**C.l.* | 0.39 | **0.043** | 0.274 |
| Anterior intestine**C.l.p.* vs Posterior intestine**C.l.p.* | 0.26 | 0.093 | 0.210 |
| Anterior intestine**C.l.p.* vs Posterior intestine**C.l.* | 0.20 | 0.162 | 0.142 |
| Anterior intestine**C.l.p.* vs Pyloric caeca**C.l.p.* | 0.05 | 1.000 | 0.758 |
| Anterior intestine**C.l.p.* vs Pyloric caeca**C.l.* | 0.47 | 0.140 | 0.488 |
| Anterior intestine**C.l.p.* vs Cardiac stomach**C.l.p.* | 0.43 | **0.035** | 0.214 |
| Anterior intestine **C.l.p.* vs Cardiac stomach**C.l.* | 0.52 | **0.035** | 0.806 |
| Anterior intestine **C.l.p.* vs Pyloric stomach**C.l.p.* | 0.46 | **0.043** | 0.530 |
| Anterior intestine **C.l.p.* vs Pyloric stomach**C.l.* | 0.46 | **0.035** | 0.459 |
| Anterior intestine **C.l.* vs Middle intestine**C.l.p.* | 0.62 | **0.035** | 0.176 |
| Anterior intestine **C.l.* vs Middle intestine**C.l.* | 0.10 | 0.510 | 0.108 |
| Anterior intestine **C.l.* vs Posterior intestine**C.l.p.* | 0.27 | **0.035** | **0.024** |
| Anterior intestine **C.l.* vs Posterior intestine**C.l.* | 0.22 | 0.063 | **0.014** |
| Anterior intestine**C.l.* vs Pyloric caeca**C.l.p.* | 0.46 | **0.043** | 0.568 |
| Anterior intestine**C.l.* vs Pyloric caeca**C.l.* | 0.14 | 0.798 | 0.136 |
| Anterior intestine**C.l.* vs Cardiac stomach**C.l.p.* | 0.21 | 0.117 | **0.039** |
| Anterior intestine**C.l.* vs Cardiac stomach**C.l.* | 0.28 | **0.035** | 0.677 |
| Anterior intestine**C.l.* vs Pyloric stomach**C.l.p.* | 0.32 | 0.053 | 0.317 |
| Anterior intestine**C.l.* vs Pyloric stomach**C.l.* | 0.17 | 0.217 | 0.220 |
| Middle intestine**C.l.p.* vs Middle intestine**C.l.* | 0.50 | **0.035** | **0.031** |
| Middle intestine**C.l.p.* vs Posterior intestine**C.l.p.* | 0.33 | 0.094 | **0.010** |
| Middle intestine**C.l.p.* vs Posterior intestine**C.l.* | 0.29 | 0.097 | **0.006** |
| Middle intestine**C.l.p.* vs Pyloric caeca**C.l.p.* | 0.09 | 0.760 | 0.167 |
| Middle intestine**C.l.p.* vs Pyloric caeca**C.l.* | 0.72 | 0.107 | 0.767 |
| Middle intestine**C.l.p.* vs Cardiac stomach**C.l.p.* | 0.51 | **0.035** | **0.020** |
| Middle intestine**C.l.p.* vs Cardiac stomach**C.l.* | 0.59 | **0.035** | 0.148 |
| Middle intestine**C.l.p.* vs Pyloric stomach**C.l.p.* | 0.54 | **0.043** | 0.052 |
| Middle intestine**C.l.p.* vs Pyloric stomach**C.l.* | 0.54 | **0.035** | **0.040** |
| Middle intestine**C.l.* vs Posterior intestine**C.l.p.* | 0.20 | 0.162 | 0.761 |
| Middle intestine**C.l.* vs Posterior intestine**C.l.* | 0.18 | 0.236 | 0.467 |
| Middle intestine**C.l.* vs Pyloric caeca**C.l.p.* | 0.37 | **0.045** | 0.462 |
| Middle intestine**C.l.* vs Pyloric caeca**C.l.* | 0.18 | 0.426 | **0.041** |
| Middle intestine**C.l.* vs Cardiac stomach**C.l.p.* | 0.20 | 0.106 | 0.857 |
| Middle intestine**C.l.* vs Cardiac stomach**C.l.* | 0.29 | **0.035** | 0.251 |
| Middle intestine**C.l.* vs Pyloric stomach**C.l.p.* | 0.31 | **0.035** | 0.455 |
| Middle intestine**C.l.* vs Pyloric stomach**C.l.* | 0.18 | 0.165 | 0.637 |
| Posterior intestine**C.l.p.* vs Posterior intestine**C.l.* | 0.06 | 0.928 | 0.508 |
| Posterior intestine**C.l.p.* vs Pyloric caeca**C.l.p.* | 0.23 | 0.236 | 0.253 |
| Posterior intestine**C.l.p.* vs Pyloric caeca**C.l.* | 0.27 | 0.236 | **0.010** |
| Posterior intestine**C.l.p.* vs Cardiac stomach**C.l.p.* | 0.26 | **0.043** | 0.850 |
| Posterior intestine**C.l.p.* vs Cardiac stomach**C.l.* | 0.32 | **0.035** | 0.132 |
| Posterior intestine**C.l.p.* vs Pyloric stomach**C.l.p.* | 0.29 | 0.068 | 0.225 |
| Posterior intestine**C.l.p.* vs Pyloric stomach**C.l.* | 0.29 | **0.035** | 0.340 |
| Posterior intestine**C.l.* vs Pyloric caeca**C.l.p.* | 0.21 | 0.236 | 0.140 |
| Posterior intestine**C.l.* vs Pyloric caeca**C.l.* | 0.23 | 0.236 | **0.008** |
| Posterior intestine**C.l.* vs Cardiac stomach**C.l.p.* | 0.26 | **0.035** | 0.467 |
| Posterior intestine**C.l.* vs Cardiac stomach**C.l.* | 0.30 | **0.035** | 0.080 |
| Posterior intestine**C.l.* vs Pyloric stomach**C.l.p.* | 0.27 | 0.075 | 0.124 |
| Posterior intestine**C.l.* vs Pyloric stomach**C.l.* | 0.26 | **0.035** | 0.216 |
| Pyloric caeca**C.l.p.* vs Pyloric caeca**C.l.* | 0.53 | 0.236 | 0.211 |
| Pyloric caeca**C.l.p.* vs Cardiac stomach**C.l.p.* | 0.38 | **0.045** | 0.313 |
| Pyloric caeca**C.l.p.* vs Cardiac stomach**C.l.* | 0.47 | **0.045** | 0.840 |
| Pyloric caeca**C.l.p.* vs Pyloric stomach**C.l.p.* | 0.41 | 0.093 | 0.794 |
| Pyloric caeca**C.l.p.* vs Pyloric stomach**C.l.* | 0.41 | **0.045** | 0.667 |
| Pyloric caeca**C.l.* vs Cardiac stomach**C.l.p.* | 0.21 | 0.379 | **0.021** |
| Pyloric caeca**C.l.* vs Cardiac stomach**C.l.* | 0.34 | 0.090 | 0.174 |
| Pyloric caeca**C.l.* vs Pyloric stomach**C.l.p.* | 0.30 | 0.192 | 0.076 |
| Pyloric caeca**C.l.* vs Pyloric stomach**C.l.* | 0.28 | 0.140 | 0.072 |
| Cardiac stomach**C.l.p.* vs Cardiac stomach**C.l.* | 0.21 | 0.081 | 0.158 |
| Cardiac stomach**C.l.p.* vs Pyloric stomach**C.l.p.* | 0.12 | 0.430 | 0.316 |
| Cardiac stomach**C.l.p.* vs Pyloric stomach**C.l.* | 0.22 | 0.117 | 0.456 |
| Cardiac stomach**C.l.* vs Pyloric stomach**C.l.p.* | 0.26 | **0.043** | 0.583 |
| Cardiac stomach**C.l.* vs Pyloric stomach**C.l.* | 0.13 | 0.361 | 0.443 |
| Pyloric stomach**C.l.p.* vs Pyloric stomach**C.l.* | 0.30 | **0.035** | 0.829 |
