# Supplementary material for: The effect of diet on the structure of gut bacterial community of sympatric pair of whitefishes (Coregonus lavaretus): one story more
Source: PeerJ. 2019 Dec 3;7:e8005. doi: 10.7717/peerj.8005 (PMC6896945; doi:10.7717/peerj.8005)
Supplement: Table S7 — textyen C.l.–C. l. pidschian; C.l.p.–C. l. pravdinellus [file peerj-07-8005-s013.docx]

| **Comparison** | **ADONIS** | | **Homogeneity of multivariate dispersions** |
| --- | --- | --- | --- |
|  | **R^2^** | **FDR P-value** | **Permuted P-value** |
| ^¥^Anterior intestine**C.l.p.* vs Anterior intestine* *C.l.* | 0.52 | 0.088 | **0.001** |
| Anterior intestine**C.l.p.* vs Middle intestine* *C.l.p.* | 0.19 | 0.536 | **0.014** |
| Anterior intestine**C.l.p.* vs Middle intestine* *C.l.* | 0.60 | 0.125 | **0.010** |
| Anterior intestine**C.l.p.* vs Posterior intestine**C.l.p.* | 0.21 | 0.439 | **0.024** |
| Anterior intestine**C.l.p.* vs Posterior intestine**C.l.* | 0.62 | 0.125 | **0.015** |
| Anterior intestine**C.l.p.* vs Cardiac stomach**C.l.p.* | 0.93 | 0.125 | 0.075 |
| Anterior intestine**C.l.p.* vs Cardiac stomach**C.l.* | 0.77 | 0.125 | **0.006** |
| Anterior intestine**C.l.p.* vs Pyloric stomach**C.l.p.* | 0.90 | 0.125 | 0.057 |
| Anterior intestine**C.l.p.* vs Pyloric stomach**C.l.* | 0.74 | 0.088 | **0.001** |
| Anterior intestine**C.l.* vs Middle intestine**C.l.p.* | 0.37 | 0.088 | 0.057 |
| Anterior intestine**C.l.* vs Middle intestine**C.l.* | 0.09 | 0.751 | 0.926 |
| Anterior intestine**C.l.* vs Posterior intestine**C.l.p.* | 0.38 | 0.088 | 0.117 |
| Anterior intestine**C.l.* vs Posterior intestine**C.l.* | 0.18 | 0.348 | 0.421 |
| Anterior intestine**C.l.* vs Cardiac stomach**C.l.p.* | 0.45 | 0.125 | **0.003** |
| Anterior intestine**C.l.* vs Cardiac stomach**C.l.* | 0.28 | 0.125 | **0.016** |
| Anterior intestine**C.l.* vs Pyloric stomach**C.l.p.* | 0.51 | 0.088 | **0.003** |
| Anterior intestine**C.l.* vs Pyloric stomach**C.l.* | 0.26 | 0.088 | **0.001** |
| Middle intestine**C.l.p.* vs Middle intestine**C.l.* | 0.43 | 0.125 | 0.202 |
| Middle intestine**C.l.p.* vs Posterior intestine**C.l.p.* | 0.08 | 0.900 | 0.881 |
| Middle intestine**C.l.p.* vs Posterior intestine**C.l.* | 0.41 | 0.243 | 0.459 |
| Middle intestine**C.l.p.* vs Cardiac stomach**C.l.p.* | 0.72 | 0.125 | 0.117 |
| Middle intestine**C.l.p.* vs Cardiac stomach**C.l.* | 0.57 | 0.125 | 0.791 |
| Middle intestine**C.l.p.* vs Pyloric stomach**C.l.p.* | 0.73 | 0.125 | 0.098 |
| Middle intestine**C.l.p.* vs Pyloric stomach**C.l.* | 0.57 | 0.088 | 0.576 |
| Middle intestine**C.l.* vs Posterior intestine**C.l.p.* | 0.44 | 0.125 | 0.279 |
| Middle intestine**C.l.* vs Posterior intestine**C.l.* | 0.17 | 0.818 | 0.579 |
| Middle intestine**C.l.* vs Cardiac stomach**C.l.p.* | 0.54 | 0.125 | 0.066 |
| Middle intestine**C.l.* vs Cardiac stomach**C.l.* | 0.34 | 0.125 | 0.136 |
| Middle intestine**C.l.* vs Pyloric stomach**C.l.p.* | 0.58 | 0.125 | **0.032** |
| Middle intestine**C.l.* vs Pyloric stomach**C.l.* | 0.29 | 0.088 | **0.034** |
| Posterior intestine**C.l.p.* vs Posterior intestine**C.l.* | 0.39 | 0.125 | 0.579 |
| Posterior intestine**C.l.p.* vs Cardiac stomach**C.l.p.* | 0.70 | 0.125 | 0.151 |
| Posterior intestine**C.l.p.* vs Cardiac stomach**C.l.* | 0.57 | 0.125 | 0.716 |
| Posterior intestine**C.l.p.* vs Pyloric stomach**C.l.p.* | 0.72 | 0.125 | 0.153 |
| Posterior intestine**C.l.p.* vs Pyloric stomach**C.l.* | 0.58 | 0.088 | 0.512 |
| Posterior intestine**C.l.* vs Cardiac stomach**C.l.p.* | 0.57 | 0.125 | 0.094 |
| Posterior intestine**C.l.* vs Cardiac stomach**C.l.* | 0.30 | 0.125 | 0.323 |
| Posterior intestine**C.l.* vs Pyloric stomach**C.l.p.* | 0.60 | 0.125 | 0.069 |
| Posterior intestine**C.l.* vs Pyloric stomach**C.l.* | 0.23 | 0.125 | 0.127 |
| Cardiac stomach**C.l.p.* vs Cardiac stomach**C.l.* | 0.59 | 0.125 | **0.038** |
| Cardiac stomach**C.l.p.* vs Pyloric stomach**C.l.p.* | 0.25 | 0.439 | 0.478 |
| Cardiac stomach**C.l.p.* vs Pyloric stomach**C.l.* | 0.59 | 0.125 | **0.001** |
| Cardiac stomach**C.l.* vs Pyloric stomach**C.l.p.* | 0.62 | 0.125 | 0.079 |
| Cardiac stomach**C.l.* vs Pyloric stomach**C.l.* | 0.19 | 0.278 | 0.850 |
| Pyloric stomach**C.l.p.* vs Pyloric stomach**C.l.* | 0.64 | 0.088 | **0.006** |
